# Time course of changes in the transcriptome during russet induction in apple fruit

**DOI:** 10.1186/s12870-023-04483-6

**Published:** 2023-09-30

**Authors:** Jannis Straube, Shreya Suvarna, Yun-Hao Chen, Bishnu P. Khanal, Moritz Knoche, Thomas Debener

**Affiliations:** 1 Institute of Plant Genetics, Molecular Plant Breeding Section, Leibniz University Hannover, Herrenhäuser Straße 2, 30419 Hannover, Germany; 2 Institute of Horticultural Production Systems, Fruit Science Section, Leibniz University Hannover, Herrenhäuser Straße 2, 30419 Hannover, Germany

**Keywords:** Russeting, *Malus x domestica*, Fruit skin, Periderm, Cuticle, Transcriptome, Suberin, Lignin, Wounding

## Abstract

**Background:**

Russeting is a major problem in many fruit crops. Russeting is caused by environmental factors such as wounding or moisture exposure of the fruit surface. Despite extensive research, the molecular sequence that triggers russet initiation remains unclear. Here, we present high-resolution transcriptomic data by controlled russet induction at very early stages of fruit development. During Phase I, a patch of the fruit surface is exposed to surface moisture. For Phase II, moisture exposure is terminated, and the formerly exposed surface remains dry. We targeted differentially expressed transcripts as soon as 24 h after russet induction.

**Results:**

During moisture exposure (Phase I) of ‘Pinova’ apple, transcripts associated with the cell cycle, cell wall, and cuticle synthesis (*SHN3*) decrease, while those related to abiotic stress increase. *NAC35* and *MYB17* were the earliest induced genes during Phase I. They are therefore linked to the initial processes of cuticle microcracking. After moisture removal (Phase II), the expression of genes related to meristematic activity increased (*WOX4* within 24 h, *MYB84* within 48 h). Genes related to lignin synthesis (*MYB52*) and suberin synthesis (*MYB93*, *WRKY56*) were upregulated within 3 d after moisture removal. *WOX4* and *AP2B3* are the earliest differentially expressed genes induced in Phase II. They are therefore linked to early events in periderm formation. The expression profiles were consistent between two different seasons and mirrored differences in russet susceptibility in a comparison of cultivars. Furthermore, expression profiles during Phase II of moisture induction were largely identical to those following wounding.

**Conclusions:**

The combination of a unique controlled russet induction technique with high-resolution transcriptomic data allowed for the very first time to analyse the formation of cuticular microcracks and periderm in apple fruit immediately after the onset of triggering factors. This data provides valuable insights into the spatial-temporal dynamics of russeting, including the synthesis of cuticles, dedifferentiation of cells, and impregnation of cell walls with suberin and lignin.

**Supplementary Information:**

The online version contains supplementary material available at 10.1186/s12870-023-04483-6.

## Background

Russeting is a skin disorder in many fruit crop species, including apple [1–5]. In russeting, the cuticle and the epidermis are replaced by periderm. In many apple cultivars, russeting compromises the visual appearance of the fruit, thereby reducing market value. Furthermore, postharvest performance is impaired by the increased permeance of the skin to water vapor, which may result in increased mass loss and shriveling [6–8].

Periderm formation begins in the hypodermis in the vicinity of microcracks in the cuticle [9–11]. Microcracks are minute microscopic cracks that are limited to the cuticle and not visible to the naked eye [12–14]. They are the first visible symptoms in russeting [12, 15] and result from a mismatch between cuticle deposition on the one hand and growth stress during periods of rapid surface expansion on the other hand [16, 17]. Russeting is influenced by both environmental and genetic factors. Environmental factors include the exposure of fruit surfaces to moisture or high humidity during periods of high strain or mechanical damage [13, 14, 18–21]. There are genetic differences in the susceptibility of cultivars to russeting. Generally, cultivars with high variability in the cell sizes of the epidermis and hypodermis are most susceptible [22].

Molecular studies indicate that the downregulation of cuticle synthesis is an important factor in russeting. QTLs (quantitative trait loci) for russeting on chromosomes 2, 12 and 15 were identified in populations segregating for russet susceptibility [23, 24]. Within these QTLs, the major cuticle regulator *MdSHN3* [24] as well as the cutin/wax transporter *MdABCG11* [23] were associated with russet susceptibility in apple under field conditions. Comparisons between a russet-resistant and a fully russeted sport of ‘Golden Delicious’ demonstrated downregulation of two oxidosqualene cyclases (*MdOSC1* and *MdOSC*3) during microcracking of the cuticle. A change in triterpene content from ursan-type to lupane-type triterpenes was observed in russeted skins, together with an increase in *MdOSC5*, which is activated by MdMYB66 and to a lesser extent by MdMYB52 [25]. Furthermore, a bulk transcriptomic study on russeted and nonrusseted fruits revealed a large number of cuticle-related genes to be downregulated in russeted apple fruit skins at maturity [26]. Additionally, suberin-associated genes were highly expressed, together with a dense network of possible transcriptional regulators of the later processes of russeting (e.g., maturation of the periderm, impregnation of cell walls with suberin) [26]. The transcription factor *MdMYB93* was later identified as a major regulator of suberin synthesis [27], and *MdMYB52* was identified as a regulator of lignin synthesis [28]. In addition, investigations at the multispecies level revealed MYB9 and MYB107 to be major regulators of suberin formation in angiosperms [29]. The majority of transcription factors associated with the later processes of russeting belong to the R2R3-MYB family and, to a minor extent, to the AP2/EREBP, bHLH, C2H2, WRKY, and NAC-domain transcription factor families [25–27, 30–32]. Unfortunately, all of the above analyses were conducted on fruit at the mature stage, while russeting typically occurs during early development. In apple, russet susceptibility peaks during the first four weeks after full bloom [1, 12, 20, 33–36]. Unfortunately, only a few studies focused on this time period. We therefore used moisture treatment to induce russeting at defined developmental stages, including the period of highest russet susceptibility [19]. Khanal and coworkers [14] refined the system to specifically target early events in russet formation. This modification allowed a patch of fruit skin to be exposed to moisture (Phase I), while the remaining fruit surface stays dry and serves as a control. The moisture is then removed (Phase II), and the events occurring after moisture removal can be monitored [13, 21].

Here, we present a high-resolution transcriptomic study performed during the onset of russeting in apple. The objectives of this research were to analyze genes representative of Phase I and Phase II of moisture-induced russeting and to identify candidates with putative functions in russeting.

## Results

We induced russeting on fruits of the cultivar ‘Pinova’ in the 2018 and 2019 growing seasons by exposing fruits 21 or 31 days after full bloom (DAFB) to moisture for 12 d (Phase I, ‘12 d wet + 0 d dry’). During Phase I, the control fruit remained dry (‘12 d dry + 0 d dry’). For the subsequent Phase II, moisture was removed, and samples were taken at 1 d (‘12 d wet + 1 d dry’) to 8 d after moisture removal (‘12 d dry + 8 d dry’). The number of read pairs obtained after quality filtering and trimming of raw reads ranged from 56.6 M to 70.5 M for independent replicates. Reads mapped uniquely to the HFTH1 genome with a frequency between 82.9 and 95.5% (Table S1).

**Fig. 1:**
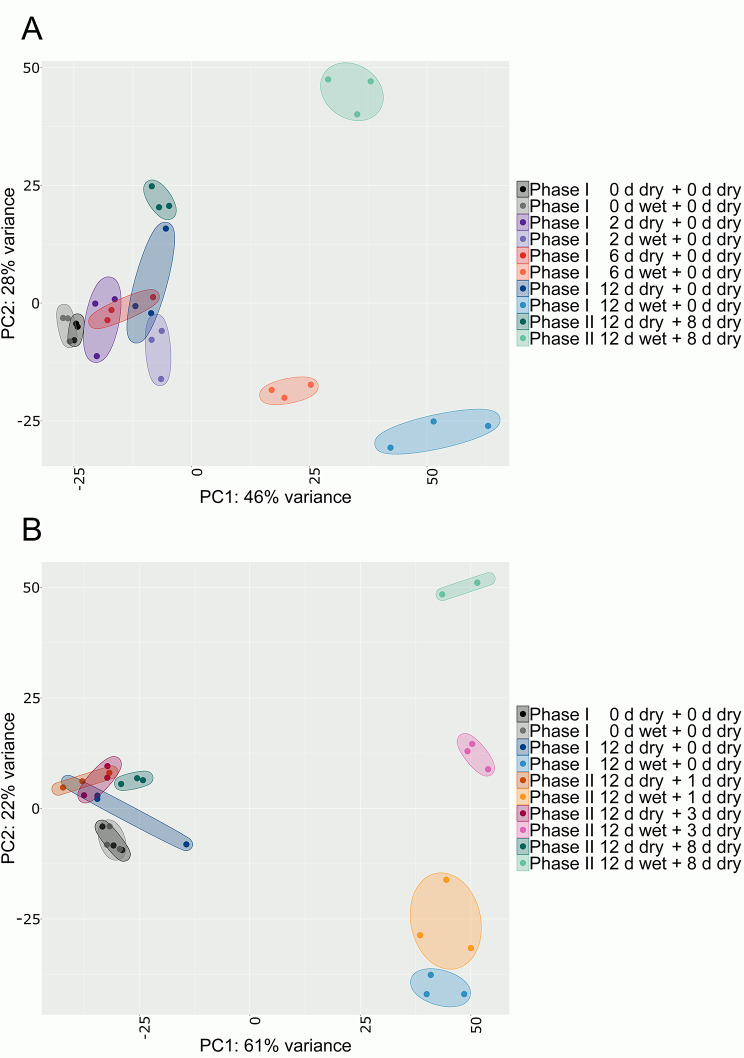
Variability between biological replicates in the RNA-Seq datasets. Apple fruit skin patches of ‘Pinova’ apples were induced to russet by exposed to surface moisture for 12 d (Phase I). After termination of moisture exposure, the treated skin patch was exposed to ambient atmosphere (Phase II). Nontreated control surfaces remained dry during Phase I and Phase II. The distribution of the transcriptome during the 2018 **(A)** and 2019 **(B)** seasons was determined by principal component analysis (PCA) based on variance stabilization transformation in ‘DESeq2’. PCA revealed clear separation of clusters between moisture-exposed (‘x d wet + y d dry’) and control (‘x d dry + y d dry’) samples, whereas the biological replicates within each treatment were consistent (indicated by ellipses)

Transcriptomic data obtained during the 2018 and 2019 seasons displayed low variability between replicates as indexed by principal component analysis (PCA) (Fig. 1A, B). Control samples clustered closely together for both seasons. In contrast, moisture-treated samples compared to untreated controls showed a pronounced diverging pattern, with distances between treatments increasing with time after moisture removal (Phase II). This corresponded to the observed progress of microcrack formation within Phase I and periderm development during the consecutive Phase II (Figure S1). Clusters observed in PCA for the 2018 season (Fig. 1A) were less compact than those in the 2019 season (Fig. 1B).

### Transcripts differ between phases I and II of russet induction

Stringent filtering of the differentially expressed genes (DEGs) obtained from the various datasets revealed a total of 3533 DEGs. The number of DEGs was higher in 2019 than in 2018 (Table S2). Four times more genes were downregulated and two times more genes were upregulated in 2019 than in 2018 between the corresponding time points at ‘12 d wet + 0 d dry’ (Phase I) and ‘12 d wet + 8 d dry’ (Phase II) (Table S2). Consistent with this observation, there were more russeted fruits in the 2019 season than in the 2018 season (Figure S2A, B).

For both seasons, we found DEGs putatively involved in microcracking in Phase I as well as in periderm formation in Phase II.

**Fig. 2:**
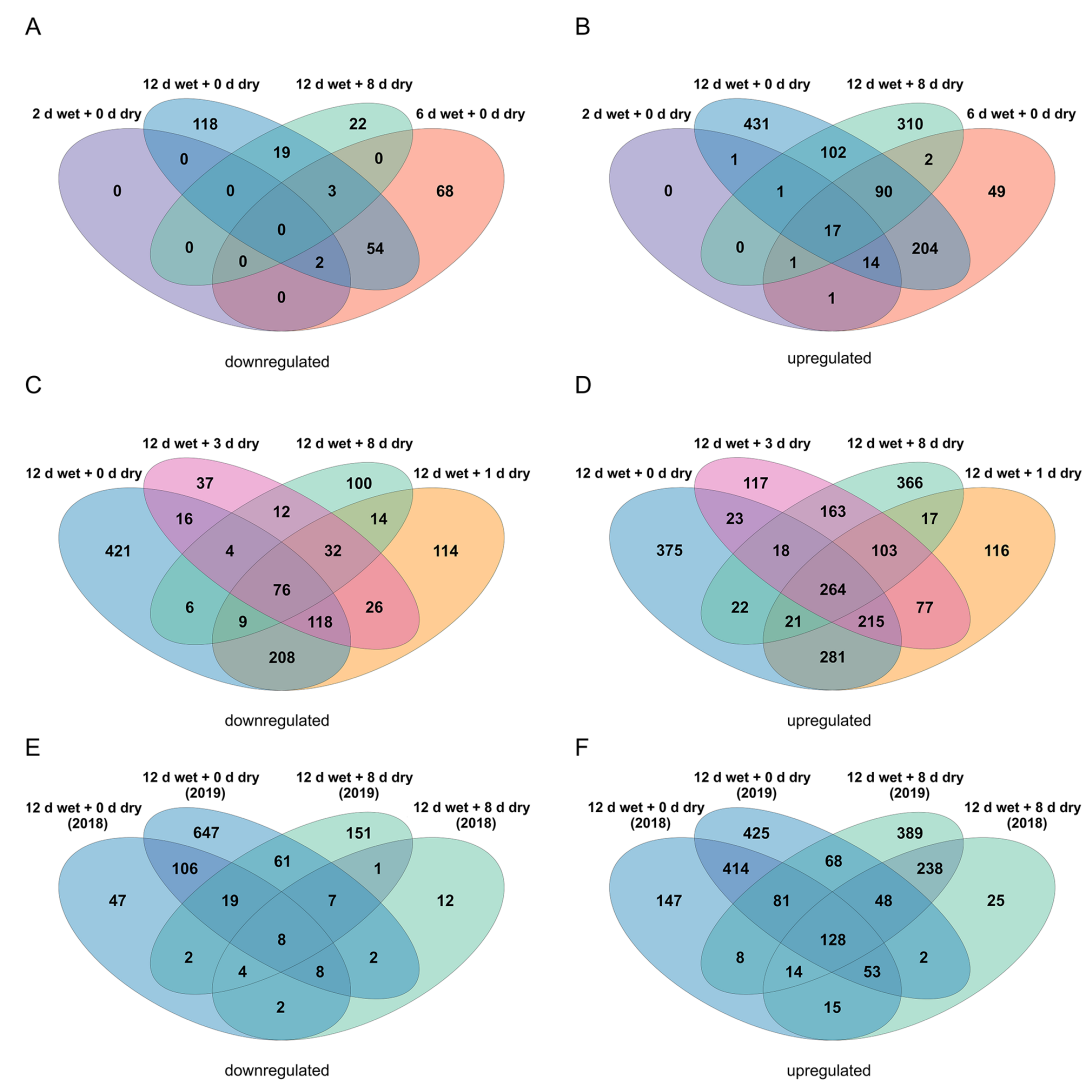
Effect of the growing season on gene expression patterns in moisture-induced russeting in ‘Pinova’ apples. Venn diagrams of differentially expressed genes during the 2018 **(A, B)** and 2019 growing seasons **(C, D)**. Comparison of common treatments and their respective controls between the two seasons **(E, F)**. Only genes with a log_2_-fold change (log_2_FC) ≥ 2 or ≤ -2, a false discovery rate (FDR) ≤ 0.05 and a mean of at least five transcripts per million (TPM) for moisture-exposed (‘x d wet + y d dry’) or control (‘x d dry + y d dry’) samples are illustrated

In Phase I samples, in the 2018 season, 242 genes were downregulated compared to 22 in Phase II. Of the 242 genes, 54 genes were downregulated at ‘6 d wet + 0 d dry’ as well as ‘12 d wet + 0 d dry’ (Fig. 2A). In contrast, 700 genes were upregulated exclusively during Phase I and 310 during Phase II. Of the 700 genes specific to Phase I, 14 were already differentially expressed after 2 d of surface moisture (‘2 d wet + 0 d dry’) (Fig. 2B).

In 2019, 421 genes were downregulated during Phase I and 335 during Phase II, whereas 32 genes were already downregulated at ‘12 d wet + 1 d dry’ in Phase II (Fig. 2C). The number of upregulated genes was 375 in Phase I and 959 in Phase II. Of the 959 genes, 103 were already upregulated at ‘12 d wet + 1 d dry’ during Phase II (Fig. 2D).

Three sampling dates were common in both seasons: ‘0 d wet + 0 d dry’ (Phase I), ‘12 d wet + 0 d dry’ (Phase I), and ‘12 d wet + 8 d dry’ (Phase II). The last two sampling times revealed a large number of season-specific DEGs. To avoid artifacts from confounding factors unrelated to russeting but differing between seasons, further analysis was restricted to DEGs consistent between seasons. These comprised 106 genes at ‘12 d wet + 0 d dry’ (Phase I) and one gene at ‘12 d wet + 8 d dry’ (Phase II) (Data S1). Eight genes were downregulated on both sampling dates (Fig. 2E). In both seasons, the number of upregulated genes was 414 at ‘12 d wet + 0 d dry’ (Phase I) and 238 at ‘12 d wet + 8 d dry’ (Phase II) (Fig. 2F; Data S1).

**Fig. 3:**
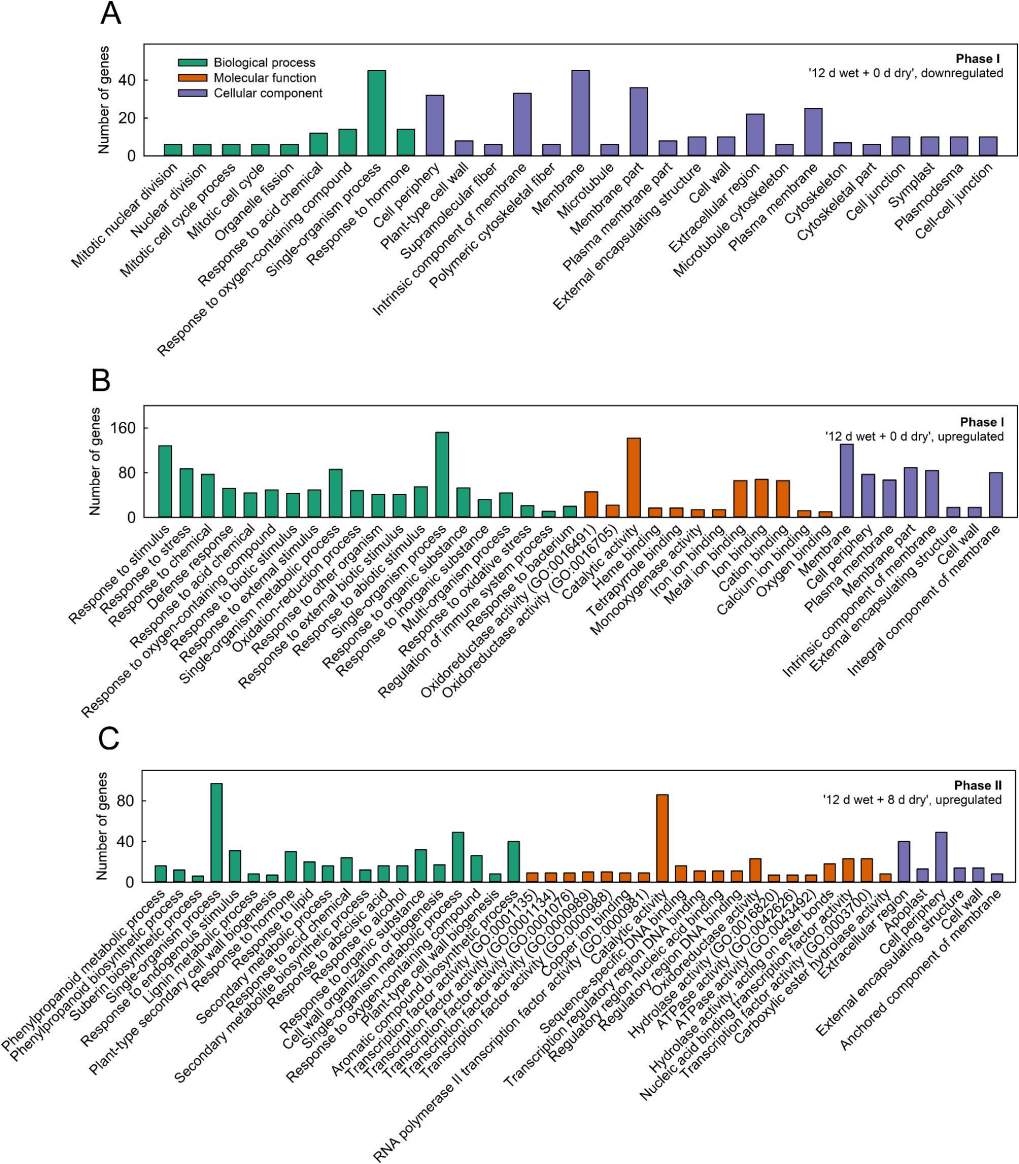
GO term analysis following moisture-induced russeting of apple. Russeting on ‘Pinova’ apple fruits was induced by moisture in a two-phase experiment. During Phase I, a patch of fruit skin was exposed to surface moisture for 12 d (Phase I). After termination of moisture exposure (Phase II), the treated skin patch was exposed to the ambient atmosphere. The nontreated controls remained dry during Phase I and Phase II. Treatments and respective controls are listed in Table S3. Moisture exposure began at 21 or 31 days after full bloom (DAFB) during the 2018 and 2019 seasons. The GO term analysis indicated weakening of the cell structure during Phase I and hormone-regulated repair mechanisms of microcracks during Phase II. Common DEGs at ‘12 d wet + 0 d dry’ **(A, B)** and ‘12 d wet + 8 d dry’ **(C)** identified by the Venn diagrams (see Fig. 2) were subjected to singular enrichment analysis (SEA) to obtain GO terms associated specifically with Phase I or Phase II. The top 20 GO terms for biological process, molecular process and cellular component are shown, which were derived from the orthologous genes found in the TAIR10 database. Only GO terms with a minimum of five genes and an FDR ≤ 0.01 were selected

The DEGs downregulated in Phase I (‘12 d wet + 0 d dry’) were characterized by gene ontology (GO) terms related to cellular processes (e.g., cell division, cell wall associated or cytoskeleton). Cuticle-related GO terms (e.g., lipid metabolic process, fatty acid metabolic process, fatty acid synthetic process, and cellular lipid metabolic process) were downregulated in 2019 and to a lesser extent (log_2_-fold change (log_2_FC) ≤ -1) in 2018 (Fig. 3A, Data S2, S3, S4, S5). The DEGs upregulated in Phase I due to moisture exposure comprised stress-related genes (e.g., oxidative stress and osmotic stress) (Fig. 3B, Data S2, S3, S4).

The DEGs during early Phase II (‘12 d wet + 1 d dry’) were similar to those at ‘12 d wet + 0 d dry’ (Phase I). The number of GO terms for downregulated DEGs decreased over time during Phase II. Beginning at ‘12 d wet + 3 d dry’, DEGs associated with suberin and lignin formation and cell wall metabolism were upregulated (Fig. 3C, Data S2, S3, S4). After ‘12 d wet + 8 d dry’ (Phase II), only one gene was consistently downregulated in both years (Fig. 2E). The upregulated genes at ‘12 d wet + 8 d dry’ (Phase II) included genes responsive to hormones, including abscisic acid (ABA), and a range of transcription factors (Fig. 3C; Data S4). At ‘12 d wet + 8 d dry’ (Phase II), processes associated with the metabolism of phenylpropanoids, suberin, and secondary metabolites, as well as response to lipids and apoplasts, were activated (Fig. 3C).

**Fig. 4:**
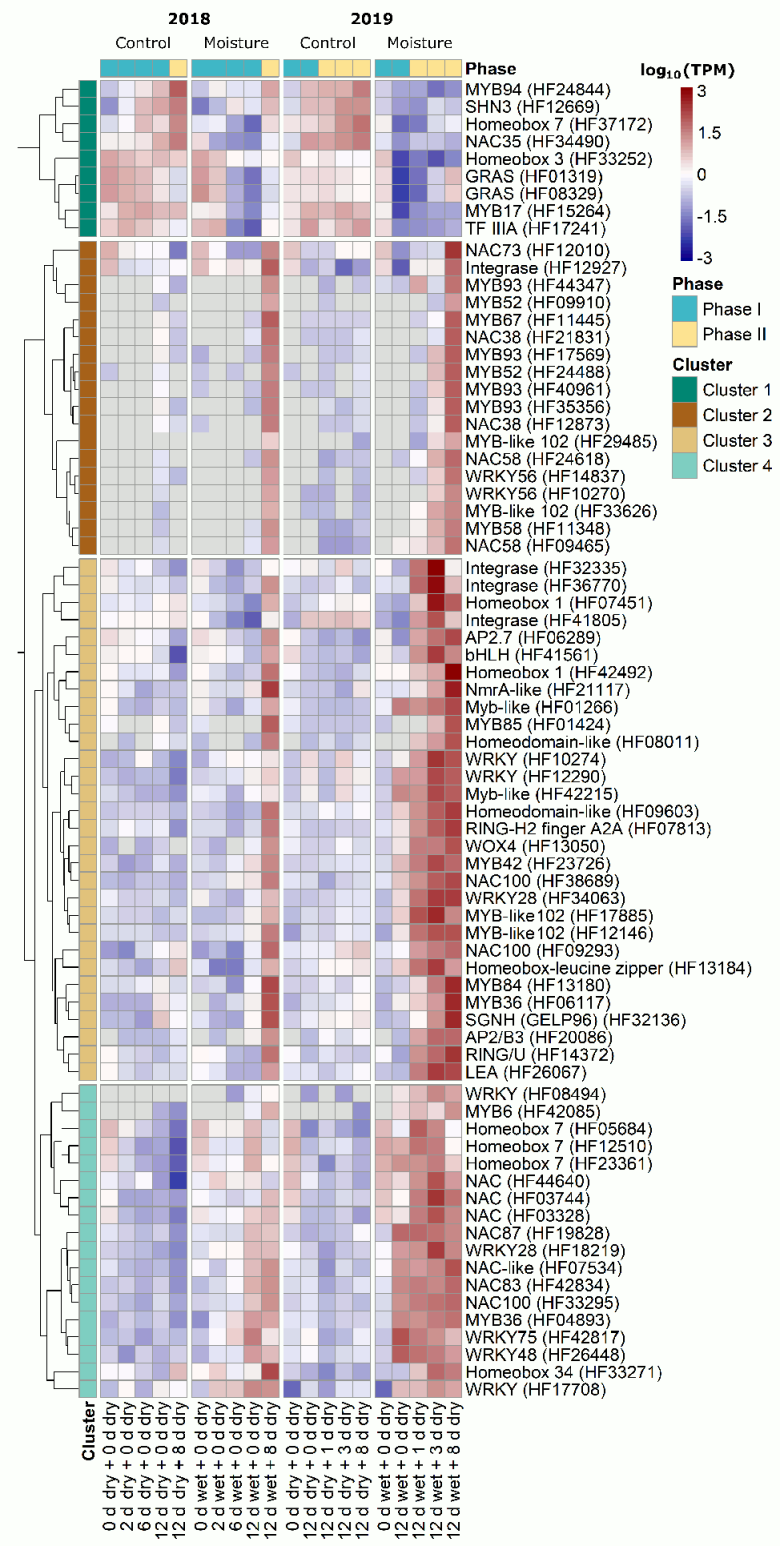
Heatmap illustrating distinct expression patterns of transcriptional regulators during moisture-induced russeting. Russeting in ‘Pinova’ apples was induced in a two-phase experiment: During Phase I, a patch of fruit skin was exposed to surface moisture for 12 d (Phase I, ‘12 d wet’). After termination of moisture exposure (Phase II), the treated skin patch was exposed to the ambient atmosphere (‘y d dry’). The nontreated control (‘Control’) remained dry during Phase I and Phase II (‘x d dry + y d dry’). The heatmap revealed a dense network of transcriptional regulators that were differentially expressed during the early phase of russet formation. Cluster 1 contains Phase I-related genes, and Clusters 2 to 4 contain Phase II-related genes. Expression values are the mean log_10_(TPM) values of three independent biological replicates comprising six (season 2018) or ten (season 2019) fruits each. Genes with a log_2_FC ≥ 2 or ≤ -2, an FDR ≤ 0.05 and a mean of at least five TPM in ‘Moisture’ or ‘Control’ at any time during the two seasons are illustrated. Gene clusters were obtained via hierarchical clustering with the R package ‘pheatmap’

Cluster analysis revealed four clusters of DEGs with highly correlated expression patterns, suggesting a close relation to the onset of russeting (Fig. 4).

The first cluster contained nine genes that were downregulated in Phase I as early as ‘2 d wet + 0 d dry’, which remained so until ‘12 d wet + 8 dry’ in Phase II. *SHN3* [37–39] and *MYB94* [40, 41] were identified within this cluster, where orthologous genes had major regulatory functions in cuticle synthesis. The second cluster contained putative regulators for russeting, e.g., *MYB93*. This gene is a major regulator of suberin formation in apple [27]. This cluster was characterized by strong upregulation (log_2_FC ≥ 2) several days after moisture removal (‘12 d wet + 3 d dry’) in Phase II. The third cluster contained transcriptional regulators that were activated immediately or shortly after termination of the moisture treatment (‘12 d wet + 0 d dry’ and ‘12 d wet + 1 d dry’) on the fruit skin patches. Within this cluster, *Wuschel-related homeobox 4* (*WOX4*) and *MYB84* were observed, which are orthologous genes of major regulators during periderm initiation. Many genes in the fourth cluster were already slightly upregulated at the timepoint of moisture removal (‘12 d wet + 0 d dry’), especially in season 2019 (Fig. 4). This cluster contains *MYB36*, the orthologous gene of which in *Arabidopsis thaliana* regulates developmental transitions from proliferation to differentiation of cells in the root endodermis [42].

### Validation of selected DEGs by qPCR

**Fig. 5:**
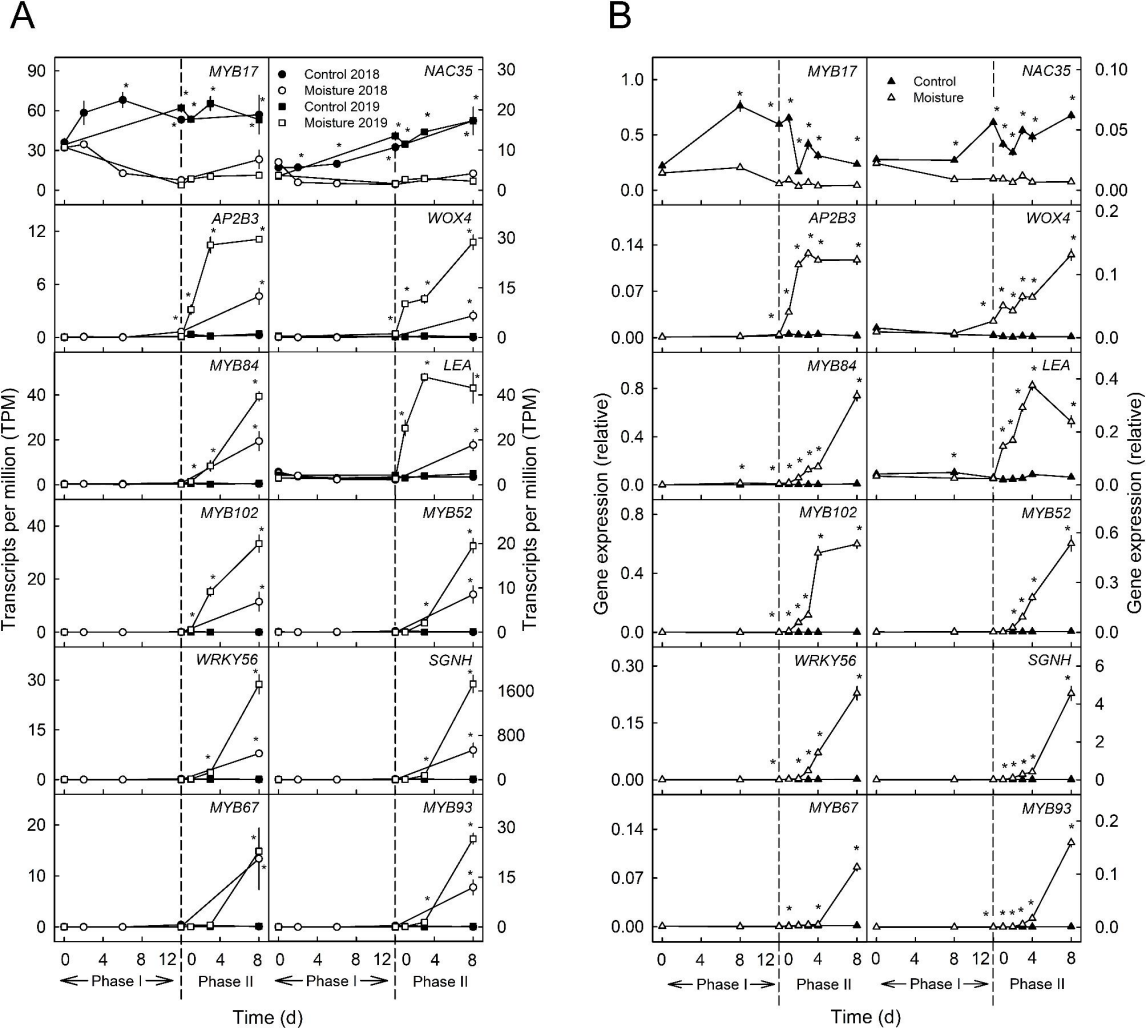
Comparison of gene expression results obtained by RNA-Seq **(A)** and qPCR **(B)**. The data obtained by the two methods reveal consistent gene expression. Russeting in ‘Pinova’ apples was induced in a two-phase experiment: During Phase I, a patch of fruit skin was exposed to surface moisture for 12 d (Phase I, ‘12 d wet’). After termination of moisture exposure (Phase II), the treated skin patch was exposed to the ambient atmosphere (‘y d dry’). The nontreated control (‘Control’) remained dry during Phase I and Phase II (‘x d dry + y d dry’). Moisture exposure began at 21 or 31 days after full bloom (DAFB) during the 2018 and 2019 seasons. The dashed line indicates the termination of moisture exposure. Genes with specific patterns for Phase I and Phase II were analyzed. Expression values obtained from RNA-Seq data **(A)** represent means ± SEs of TPM of three independent biological replicates comprising six (season 2018) or ten (season 2019) fruits each. * indicates a significant difference between ‘Moisture’’ and ‘Control’ at FDR ≤ 0.05. Expression values derived from qPCR **(B)** represent means ± SEs of three independent biological replicates comprising ten fruits each. ‘*’ indicates a significant difference between ‘Moisture’ and ‘Control’ at *p* ≤ 0.05 (Student’s t test)

Earlier studies established that (1) microcracking of the cuticle occurs within 48 h of moisture exposure and (2) periderm initiation begins within 24 h of moisture removal [13, 14, 21, 43]. Therefore, putative regulators must be expressed early during Phase I and at the beginning of Phase II, i.e., at ‘12 d wet + 1 d dry’. Based on these findings, a set of 12 DEGs was selected that represented candidate genes for early regulation during Phase I and Phase II (Fig. 5A) and/or are related to either cuticle (Phase I) or periderm formation (Phase II). These comprised transcription factors derived from clusters specific to Phase I (*MYB17*, *NAC35*) or Phase II (*AP2/B3-like transcription factor family protein* (*AP2B3*), *WOX4*, *MYB84*, *MYB-like 102* (*MYB102*), *MYB52*, *WRKY56*, *MYB67*, *MYB93*), one *late embryogenesis abundant hydroxyproline-rich glycoprotein* (*LEA*) and one *SGNH hydrolase* (*SGNH*). The genes were analyzed by quantitative real-time PCR (qPCR) to validate their expression patterns (Fig. 5B). The expression patterns of the selected genes were similar for RNA-Seq and qPCR. *MYB93* was used to trace the early processes of suberin synthesis [21, 27, 43] during Phase II and thus was representative of periderm formation as indexed by the occurrence of phellem cells.

*NAC35* and *MYB17* were downregulated during early Phase I within both seasons at time points when microcracking occurred (Fig. 5A, B; Figure S1). The genes *AP2B3*, *WOX4* and *LEA* showed increased expression at ‘12 d wet + 1 d dry’ (Fig. 5A, B). At ‘12 d wet + 2 d dry’, the expression of *MYB84*, *MYB102*, *MYB52* and *WRKY56* increased as indexed by qPCR. Upregulation of *MYB93* and *SGNH* started one day later at ‘12 d wet + 3 d dry’. The increase continued until ‘12 d wet + 8 dry’ (Fig. 5B). The expression pattern was consistent between the two seasons (Fig. 5A, B). The transcriptional regulator *MYB67* was only differentially expressed at ‘12 d wet + 8 d dry’, when the first phellem cells had formed (Fig. 5, Figure S1).

### Expression patterns of DEGs match russet susceptibility in cultivars differing in russet susceptibility

**Fig. 6:**
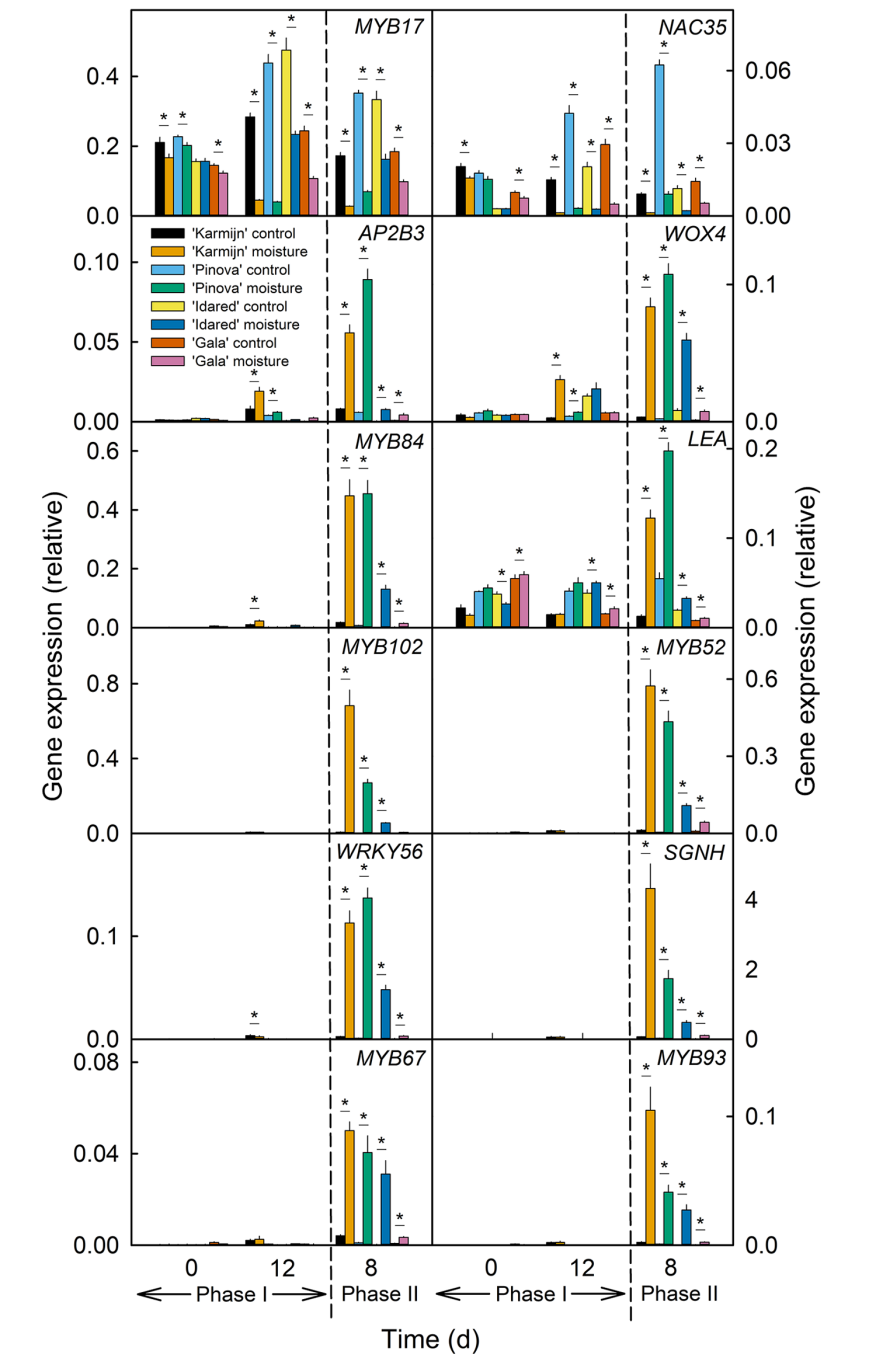
Expression pattern of putative candidate genes during moisture-induced russeting of four apple cultivars. A two phase experiment was conducted to induce russeting in four apple cultivars (‘Karmijn’, ‘Pinova’, ‘Idared’, and ‘Gala’) that vary in their susceptibility to russet: During Phase I, a patch of fruit skin was exposed to surface moisture for 12 d (Phase I, ‘12 d wet’). After termination of moisture exposure (Phase II), the treated skin patch was exposed to the ambient atmosphere (‘y d dry’). The nontreated control (‘Control’) remained dry during Phase I and Phase II (‘x d dry + y d dry’). The dashed line indicates the termination of moisture exposure. The expression of genes associated with Phase I *(MYB17*, *NAC35*) as well as Phase II (*AP2B3*, *WOX4*, *MYB84*, *LEA*, *MYB102*, *MYB52*, *WRKY56*, *SGNH*, *MYB67, MYB93*) was analyzed. Expression values represent the means ± SEs of three independent biological replicates comprising six fruits each. ‘*’ indicates a significant difference between ‘Moisture’ and ‘Control’ in each cultivar at *p* ≤ 0.05 (Student’s t test)

To confirm a role in russeting, the expression pattern of the selected DEGs was studied in four cultivars differing in russet susceptibility. Russet susceptibility decreased from ‘Karmijn’>‘Pinova’>‘Idared’>‘Gala’, as indexed by the portion of russeted surface area within the moisture-exposed skin patch [44] (Figs. S3, S4). Downregulation of the Phase I-related gene *MYB17* correlated with the degree of russet susceptibility (Fig. 6). Only for *NAC35* was there no relationship to russet susceptibility (Fig. 6).

Generally, the expression patterns of Phase II-related genes (*LEA*, *WOX4*, *AP2B3*, *MYB52*, *MYB67, MYB84*, *MYB93*, *MYB102*, *WRKY56* and *SGNH*) corresponded to the extent of microcracking during Phase I (Figure S5) and matched the degree of russet susceptibility of the four cultivars during Phase II (Fig. 6, Figure S3).

### Phase II genes display similar expression patterns in samples where russeting is induced by moisture or by mechanical wounding

Mechanical wounding of apple fruit skins induced russeting. Hence, the expression patterns of the DEGs were also analyzed following wounding [43].

**Fig. 7:**
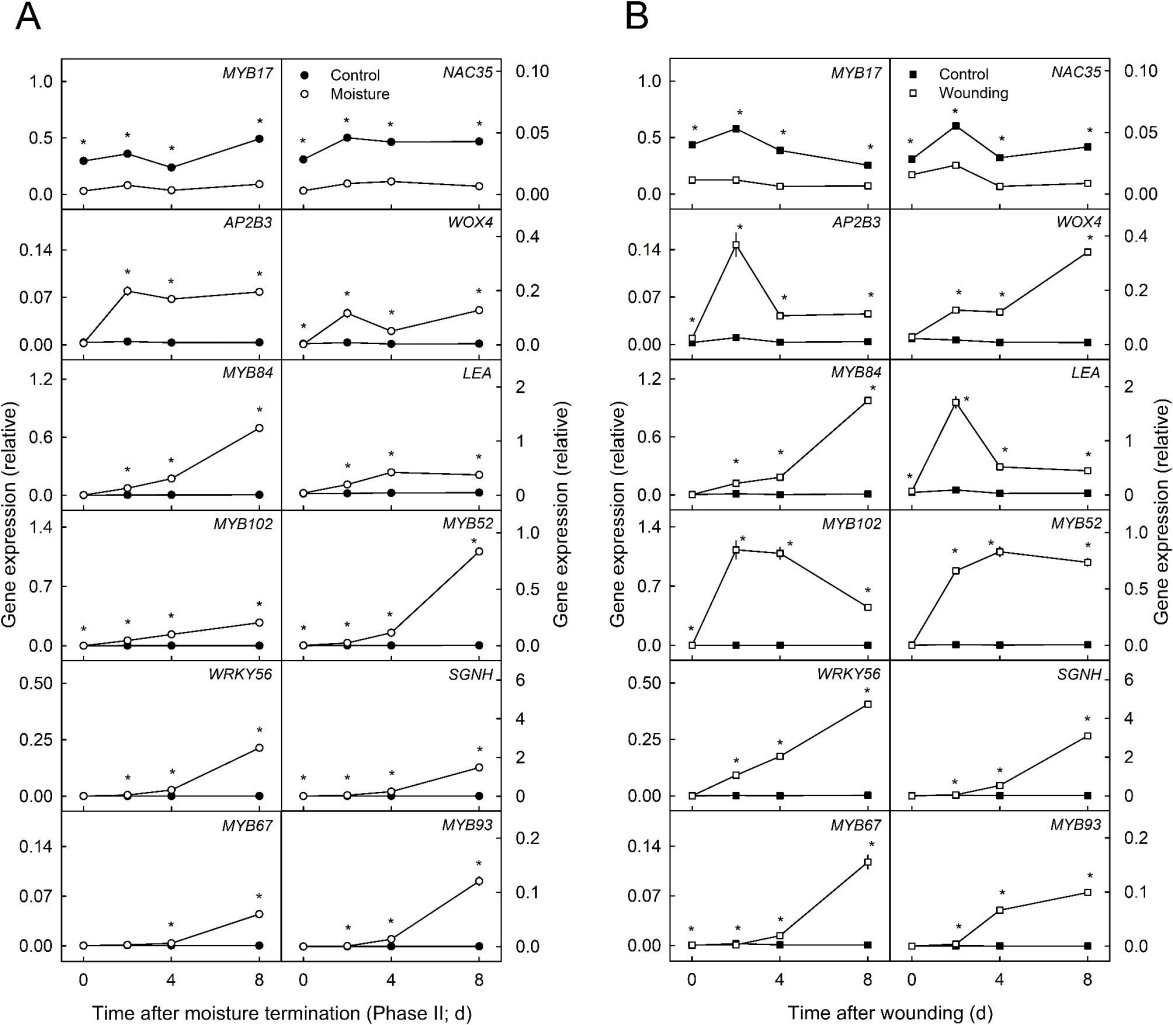
Expression of putative candidate genes involved in russeting during moisture-induced **(A)** or wound-induced **(B)** russeting. Russeting in ‘Pinova’ apples was induced by surface moisture in a two-phase experiment: During Phase I, a patch of fruit skin was exposed to surface moisture for 12 d (Phase I, ‘12 d wet’). After termination of moisture exposure (Phase II), the treated skin patch was exposed to the ambient atmosphere (‘y d dry’). The nontreated control (‘Control’) remained dry during Phase I and Phase II (‘x d dry + y d dry’). Russeting was also induced by mechanical wounding using sandpaper (‘Wounding’). The nontreated fruit skin served as a control (‘Control’). The data revealed similar expression patterns between the two types of russet induction. Gene expression of candidate genes for the onset of periderm formation was determined at 0, 2, 4 and 8 d after moisture termination **(A)** or after wounding **(B)**. Expression values represent the means ± SEs of three independent biological replicates comprising six fruits each. ‘*’ indicates a significant difference between ‘Moisture’ and ‘Control’ or ‘Wounding’ and ‘Control’ at *p* ≤ 0.05 (Student’s t test)

The Phase I-specific transcription factors *MYB17* and *NAC35* were downregulated immediately after wounding (Fig. 7).

*AP2B3*, *WOX4*, *MYB84*, *MYB102*, *MYB52*, *WRKY56* and *LEA* were upregulated 2 d after wounding, and *SGNH*, *MYB67* and the suberin-specific gene *MYB93* were upregulated after 4 d (Fig. 7). The expression of *AP2B3*, *LEA* and *MYB102* peaked at 2 d and decreased to a constant level thereafter. The expression of *MYB67* was similar to that of *MYB93* and *SGNH*, although the increase was somewhat smaller. Similar DEGs were identified in the cultivar comparison following wounding. There was no relationship between the russet susceptibility of the cultivars and the DEGs (Figure S6). In contrast to moisture-induced russeting, which induced russeting only in a fraction of the exposed skin patch, russeting following wounding covered the entire area of the wounded patch in all four cultivars (Table S4).

## Discussion

Our discussion focuses on (1) the suitability of moisture-induced microcracking for studying russeting in apples, (2) the changes in the transcriptome occurring during Phase I and (3) those occurring during Phase II of moisture-induced russeting.

### Moisture-induced russeting

Russeting in susceptible apple cultivars is triggered by a number of environmental factors [13, 14, 18–21, 36, 45–53]. Under field conditions, these factors are impossible to control, resulting in high variability of russeting within a tree, between trees, and between orchards, regions and seasons.

This makes systematic studies on russeting and the identification of triggers of russeting at a molecular level difficult. Moisture-induced russeting is a promising system that offers several advantages. First, surface moisture is a common factor in the natural russeting of apples [15, 18, 36, 54]. Second, experimental induction of russeting using moisture may be performed at the developmental stage where fruit is most susceptible to russeting. This is the first 40 days after full bloom [1, 12, 20, 33–36]. However, most studies of transcriptomes of russeted apple fruit are based on natural russeting assessed at the mature stage [24–26, 29–32]. Furthermore, the beginning of russet induction is precisely defined in moisture-induced russeting. In contrast, when assessing russeting at the mature stage, the time of the onset of russeting is unknown. This makes it impossible to establish causal relationships between potential trigger(s) of russeting. Third, our system of moisture-induced russeting allows us to compare the transcriptomes of the nonrusseted control and the russet-induced skin patch on an individual fruit basis. Thus, differential gene expression is standardized for differences between cultivars, stages of fruit development and environmental factors to which the fruit is exposed in the tree canopy. This is not the case when susceptible and resistant cultivars are compared. In the latter case, differences between cultivars and the specific environment of the fruit cannot be separated from genetic differences in russet susceptibility. These arguments demonstrate that moisture-induced russeting offers a high degree of control. The system was successfully used previously [13, 14, 21, 43]. The data obtained demonstrate that during Phase I within 48 h of moisture exposure, cuticle synthesis decreases, and microcracks are formed. The microcracks represent the first visible symptoms of russeting [10, 12, 15]. During the subsequent Phase II after moisture removal, microcracks are exposed to the ambient atmosphere, and the periderm begins to differentiate [13, 21, 43]. Stringent filtering of DEGs combined with high sequencing depth (59.8 to 75.1 million read pairs per biological replicate) allowed us to also identify lowly expressed genes such as transcription factors relevant to russeting. Furthermore, performing the experiment in different growing seasons allowed us to identify consistent changes between the two seasons. This comparison demonstrated a remarkable degree of overlap that was further confirmed by qPCR of selected genes in subsequent seasons. In addition, Phase II processes were consistently altered in the wounding treatments. Like exposure to surface moisture, the developmental stage of the wounding treatment is well defined, and the treatment is performed during the phase of maximum susceptibility to russeting. Based on these arguments, the induction of russeting by moisture exposure or wounding is a helpful tool in identifying triggers of russeting.

### Genes differentially expressed in phase I

Phase I of russet induction was characterized by a large number of downregulated genes related to either cutin and wax synthesis (*SHN3* [37], *GPAT6* [55], *WSD1* [56], *ABCG11* [57], *LTP3* [30]), transcriptional regulation (*MYB17*, *NAC35*) or cell cycle and microtubule formation (*Tubulin/FTsZ family protein* (HF08104) and *ATP binding microtubule motor family protein* (HF30539)). These data are consistent between RNA-seq and qPCR. They also confirm the findings of earlier studies on cutin and wax deposition in relation to moisture-induced russeting [21, 43].

The downregulation of genes involved in cuticle formation is considered to be an early factor associated with microcracking during Phase I [23–26, 30]. We therefore compared the transcriptional dynamics of these genes to those of our new set of candidate genes in our RNA-Seq dataset (Figure S7). Cuticle-related genes decreased during Phase I after 6 d of moisture exposure (‘6 d wet + 0 d dry’) in 2018 and after 12 d (‘12 d wet + 0 d dry’) in both seasons (Figure S7). Several regulators in Cluster 1 (Fig. 4) were downregulated, including *homeobox 7*, *NAC35*, two *GRAS transcription factors*, *MYB17*, and *TF IIIA*. These were downregulated before the cuticle-related genes *SHN3*, *ABCG11*, *GPAT6*, *KCS10*, *WSD1* and *CER6* (Figure S7) were downregulated during Phase I.

The transcription factors *MYB17* and *NAC35* were the earliest genes downregulated after the beginning of the moisture treatments. The expression pattern of *MYB17* correlated closely with that of *SHN3* [24, 38, 39]. However, the downregulation of *MYB17* occurred slightly earlier during Phase I as well as to a greater extent. *MYB17* is highly similar to *AtMYB16* and *AtMYB106.* The last two are involved in the regulation of epidermal cell growth and cuticle formation [58, 59]. A putative role of *MYB17* in cuticle formation is also consistent with the cultivar comparison of moisture-induced russeting (Fig. 6) and the experiment on wound-induced russeting (Figure S6). Here, the expression of *MYB17* was much lower in susceptible cultivars than in resistant cultivars. Wounding resulted in decreased expression of *MYB17*.

The second transcription factor, *NAC35*, was chosen because overexpression of *AtLOV1*, an ortholog of *NAC35* in *Arabidopsis thaliana*, changed epidermal cell organization and increased lignin content in cell walls when overexpressed in switchgrass [60]. The *MYB17* expression patterns of NAC35 were consistent between the qPCR experiments and the RNA-Seq analysis.

The expression of *MYB17* and *NAC35* after moisture treatment was also confirmed in a fourth experiment in which russet induction by wounding and moisture was compared. In both treatments, *MYB17* and *NAC35* were downregulated. This downregulation is in line with that of other cuticle-specific genes, such as *SHN3, GPAT6*, *KCS10*, *WSD1*, *CER6* and *ABCG11*, described in earlier studies [43]. However, their differential regulation after mechanical wounding indicates that the downregulation is not related to microcracking typical of Phase I of russet induction but rather to the tissue damage that accompanies skin cracking.

Genes upregulated during Phase I were stress response genes such as *1-aminocyclopropane-1-carboxylic acid (acc) synthase 6* (HF20852), *peroxidase superfamily protein* (HF39739), and *heat shock protein 70* (HF0032) and genes related to oxidative stress, osmotic stress and salt stress. There were no upregulated genes that are involved in periderm formation.

Interestingly, the regulation of genes as indexed by the log_2_FC in expression was larger in the 2019 than in the 2018 growing season. This was consistent with more severe russeting in 2019 than in 2018, probably as a result of seasonal differences in temperature and rainfall (Supplementary data, [13]) (Figure S2).

The mechanism of moisture-induced microcracking of the cuticle is probably related to failure of the hydrated cuticle when exposed to growth stress and strain. Cuticle hydration decreases the fracture force, which facilitates microcracking [61]. Additionally, the growth strain is particularly high during early fruit development, when the growth rate in surface area is high relative to the surface area present at that time (Figure S8). Importantly, the mechanical properties of the cuticle do not differ between russet-susceptible and nonsusceptible cultivars [44]. In apple, the epidermis and hypodermal cell layers form the structural backbone of the fruit skin [62]. Russet-susceptible cultivars differ from nonsusceptible cultivars in that they have a higher variability of cell sizes in the epidermis and hypodermis [22]. Variable and larger cell sizes of the fruit skin cause stress concentration and failure when the skin is strained. During this process, the cuticle is dragged along and fails in response to the underlying cells [63]. Based on the above arguments, the change in mechanical properties of the cuticle and the decrease in cuticle deposition as a result of the downregulation of genes involved in cuticle formation during a phase of high growth stress are causal in failure. Variable cell sizes predispose fruit skins to russeting.

### Genes differentially regulated during phase II

Periderm formation occurs during Phase II. It requires the differentiation of a periderm in hypodermal cell layers underneath an epidermis with a microcracked cuticle. Periderm formation is a three-step process comprising (1) the formation of a meristem, the phellogen, that (2) then begins to divide to produce stacks of phellem cells. The final step in periderm formation (3) is the incrustation of the phellem cell walls with suberin and lignin. Earlier studies established that in moisture-induced russeting, this three-step process begins in Phase II only after removal of moisture when the treated skin patch is exposed to the ambient atmosphere [13, 21], irrespective of the duration of moisture exposure. Our findings are consistent with this conclusion.

Based on the above arguments, during **the early Phase II, differentially expressed genes should comprise genes characteristic of meristematic tissue.** This was indeed the case. Differentially expressed genes included various MYB, NAC, WRKY, and homeobox transcription factors, *WOX4*, *AP2/B3* and several LEAs, expansins, laccases and peroxidases (Figure S9, Data S6, S7). The expression of these genes increased immediately after moisture removal and exposure of the skin patch to the ambient atmosphere. Many of these genes are related to periderm formation (Figs. 4, [25, 30, 64–68]).

Recently published studies suggested that genes encoding proteins with acyltransferase or esterase/lipase activity, cell wall metabolism, pentacyclic triterpene synthesis, the phenylpropanoid pathway, suberin synthesis and transport of lipids are possible candidates in russeting [23, 25, 26, 30]. The cell wall-associated genes xyloglucan endotransglucosylases/hydrolases (XTH), expansins (EXP), peroxidases (PRX) and *laccase 7* (*LAC7*) increased in moisture-exposed patches during the transition from Phase I to Phase II at ‘12 d wet + 0 d dry’. Three acyltransferases associated with triterpene-hydroxycinnamates as well as several genes associated with esterases/lipases (GDSL), pentacyclic triterpene synthesis, suberin synthesis, phenylpropanoid synthesis and lipid transport increased in gene expression at ‘12 d wet + 3 d dry’ or afterward (Figure S9). Transcriptional regulators found within Clusters 3 and 4 (Fig. 4) were upregulated earlier than most of the genes associated with phenylpropanoid or suberin synthesis (Figure S9), while genes found in Cluster 2 showed expression patterns similar to those of suberin-associated genes.

A total of four genes (*WOX4, AP2B3, LEA*, and *MYB84*) were validated by qPCR. The increase in expression was consistent between qPCR and RNA-Seq and occurred within 24 h (*WOX4, AP2B3, LEA*) and 48 h (*MYB84*) after exposure to the ambient atmosphere. Furthermore, the expression of all four genes was markedly higher in russet-susceptible cultivars than in nonsusceptible cultivars, implying a role in russeting.

The ortholog of *WOX4* in *Arabidopsis* [69–72] and poplar [73] is related to the formation of the vascular cambium. In moisture-induced russeting in apple, *WOX4* was among the earliest expressed genes in Phase II. In the 2019 and 2020 seasons (cultivar comparison), it was already expressed to some extent late in Phase I (Figs. 5 and 6). The upregulation, however, was restricted to cultivars of high susceptibility in Phase I (Fig. 6). In Phase II, *WOX4* was more regulated in susceptible than in resistant cultivars. Interestingly, *WOX4* was also expressed after mechanical wounding (Fig. 7, Figure S6). These arguments suggest that *WOX4* is a candidate gene for phellogen formation.

An ortholog of *AP2B3* in *Arabidopsis*, *AtNGA1*, regulates 9-cis-epoxycarotenoid dioxygenase 3 (AtNCED3), which is involved in ABA formation upon drought stress [74]. *AP2B3* was induced even earlier than *WOX4* (Fig. 5). The function of *AP2B3* is consistent with its expression during early Phase II. Moisture removal after Phase I increased water loss from the microcracked cuticle – the microcracks shunted the barrier properties of the cuticle [63]. The water loss, in turn, induced drought stress. In line with this, we found an ortholog of *NCED3* in apple (HF22773) that was differentially expressed in Phase II. *AP2B3* expression was also reported in russeted fruit at later developmental stages [25].

The early induction of LEA is consistent with the above arguments (Figs. 5 and 7). LEA proteins are known to be responsive to ABA and are enriched in response to abiotic stress, including drought [75]. The *LEA* gene in our study is an ortholog of *AtNHL26*, which is active within the phloem [76].

The DEG *MYB84* is an ortholog of *MYB1* of *Quercus suber*, where it is specific to phellem cells [66, 67]. Additionally, in *Arabidopsis* hypocotyls and roots, *MYB84*/*RAX3* are expressed in the periderm [65]. These arguments are consistent with a role of *MYB84* in the formation of the phellogen.

During the **later Phase II of periderm formation, we expect differential expression of genes related to the incrustation of cell walls with suberin and lignin.** This was confirmed in our experiment. The GO term analysis of the differentially expressed genes identified genes involved in suberin, phenylpropanoid and lignin metabolism and synthesis, genes involved in ABA metabolism and genes related to cell wall synthesis (Fig. 3). In addition, a number of transcription factors belonging to the MYB, WRKY and NAC families were found to be solely expressed during late Phase II (Fig. 4).

We selected six genes (*MYB93, MYB102*, *MYB52*, *WRKY56*, *SGNH*, and *MYB67*) with putative functions in suberin formation for further validation by qPCR. Again, the expression patterns obtained by qPCR and RNA-Seq were consistent. The increased expression of *MYB93* was consistent with that obtained in earlier studies [21, 43]. Its expression pattern perfectly mirrored the differential russet susceptibility in the cultivar comparison (Fig. 6). Additionally, the expression of *MYB93* after wounding further supported a role in russeting. In response to mechanical wounding, a periderm was induced after four days, which then began to divide to produce phellem [43]. The suberization of the cell wall is consistent with the expression of *MYB93*. *MYB93* has been reported to be involved in suberization of russet periderm [27]. *MYB93* was also reported to interact with other genes. When overexpressed in *N*. *benthamiana* leaves, *MYB93* induced the expression of *MYB52*, *MYB67*, *WRKY56* and *MYB84*, the last to a slightly lower extent.

Similar to *MYB93, MYB102* is another interesting candidate for periderm formation during late Phase II. The expression of its ortholog *AtMYB102* in *Arabidopsis* is directly induced by ABA. In *Arabidopsis thaliana*, ABA increased the suberization of roots [77]. Furthermore, *MYB102* responded to wounding [78], which is consistent with its role in the late phase of periderm formation.

Similarly, *AtGELP96*, an ortholog of *SGNH*, has key functions in the polymerization of suberin together with four other GELPs (*GELP22*, *GELP38*, *GELP49*, and *GELP51*) in *A*. *thaliana* roots [79]. This finding supports the putative functions of these genes in the accumulation of suberin in phellem cells after the phellogen has developed. Both the expression pattern and the annotations of *MYB52*, *MYB67*, *MYB102* and *WRKY56* indicated that these genes also contributed to the differentiation of the developing periderm during late Phase II rather than the development of the phellogen. *MYB52*, *MYB67*, *MYB102* and *WRKY56* were all induced at later stages of periderm formation. Their expression patterns were highly correlated with the extent of russeting in the cultivar comparison (Fig. 6).

## Conclusion

**Fig. 8:**
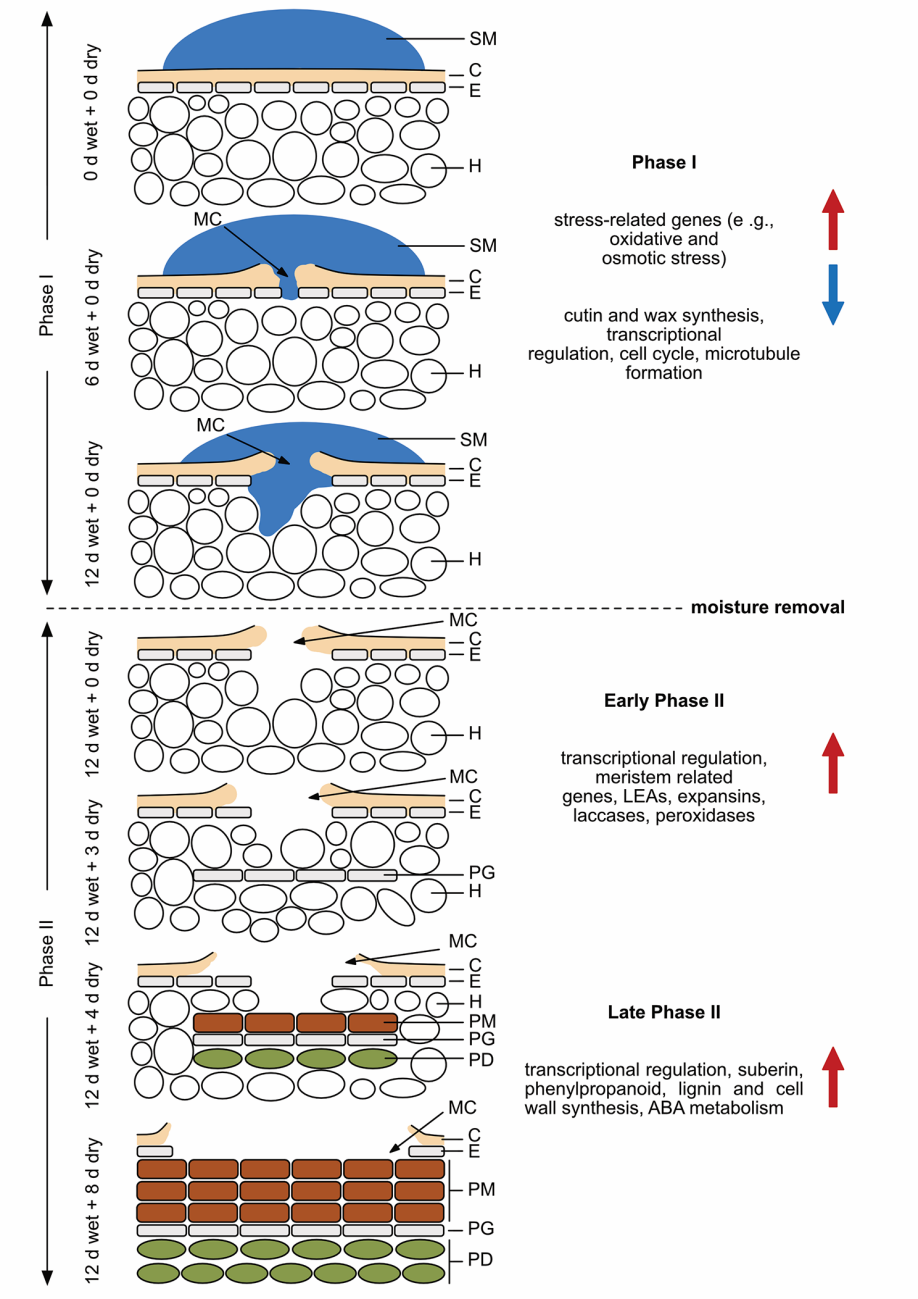
Sketch of sequence of events in moisture-induced russeting of apple fruit skins. In Phase I, the skin patch is exposed to moisture for 12 d during early fruit development (21–31 days after full bloom (DAFB)). In Phase II, the moisture is removed and the fruit surface exposed to atmospheric conditions. In Phase I microcracks in the cuticle are detected as early as 2 d of moisture exposure. Over time, these microcracks expand tangentially and radially. They traverse the cuticle radially by day 6 of moisture exposure. As the fruit enters Phase II, meristem-related genes are activated indicating the formation of a phellogen in the hypodermis underneath a microcrack (0–3 d after moisture removal). During the late stage of Phase II (starting 3–4 d after moisture removal), the phellogen differentiates a phelloderm and produces suberized phellem cells. By 8 d after moisture exposure, a continuous periderm has developed. Gene groups that are up-regulated during each phase (Phase I, early Phase II, and late Phase II) are marked by a red arrow on the right side of the panel. Conversely, gene groups that are downregulated during these phases are indicated by a blue arrow. SM = surface moisture, C = cuticle, E = epidermis, H = hypodermis, MC = microcrack, PG = phellogen, PM = phellem, PD = phelloderm.

The analysis of the transcriptome during periderm formation revealed a distinct pattern of gene expression. Based on the expression profiles and the supposed functions in heterologous plant systems, the following sequence of events results in periderm formation and, hence, russeting (Fig. 8). The downregulation of genes involved in cutin and wax synthesis and deposition and the simultaneous change in the mechanical properties of the cuticle due to hydration result in microcrack formation during moisture exposure. After moisture removal, the tissue underneath the microcracks comes into contact with the ambient atmosphere. A cascade of transcriptional regulatory events is now initiated. The increase in transpiration caused by the impaired barrier properties of the cuticle locally induces water stress as indexed by the expression of stress-related genes. At the same time, a yet unknown trigger induces the differentiation of the phellogen, as indexed by the expression of genes related to meristematic activity during early Phase II. The subsequent incrustation of the phellem with suberin and lignin (late Phase II) is consistent with the expression of genes involved in suberin and lignin synthesis and the regulation thereof. Notably, the differentially expressed genes identified in the transcriptomic analysis of the developmental time course during Phase II were also observed in the comparison of cultivars varying in russet susceptibility and the response to mechanical wounding.

This study provides transcriptomic resources for early events of artificially induced russeting in apple and further data on the comparison of mechanically induced versus moisture-induced russeting in terms of the expression of selected genes, which may help finally identify the molecular triggers of russet induction.

## Materials and methods

### Plant materials

Apple fruits (*Malus x domestica* Borkh.) of ‘Karmijn’, ‘Pinova’, ‘Idared’ and ‘Gala’, all grafted on M9 rootstocks, were cultivated in experimental orchards of the horticultural research station of the Leibniz University Hanover at Ruthe (52° 14’ N, 9° 49’ E). These cultivars differ in susceptibility to russeting in the order ‘Karmijn’>‘Pinova’>‘Idared’>‘Gala’ [44] (Figs. S3, S4).

A total of four experiments were conducted. First, the time course of change in the transcriptome was investigated in moisture-induced russeting in ‘Pinova’ using RNA-Seq and validated via qPCR. Samples were taken from a total of 125 trees. Second, gene expression in moisture-induced russeting was investigated in four cultivars differing in russet susceptibility using qPCR. The number of trees sampled was 30 per cultivar. Third, gene expression in wounding-induced russeting was investigated in four cultivars differing in russet susceptibility using qPCR. The number of trees sampled was 20 per cultivar. Fourth, gene expression was compared between moisture- and wounding-induced russeting in ‘Pinova’ using qPCR. Here, the number of trees was 125. Experiments were performed in four different growing seasons (Table S3, Figure S10).

### Russet induction

Russeting was induced either by moisture exposure or by mechanical wounding [13, 14, 21, 43]. For moisture exposure, two-phase experiments were conducted (Figure S11). Apple fruits 10–12 mm in diameter (21–32 DAFB) were selected (Table S3, [13, 14, 21, 43]). The tip of a 2.0 ml polyethylene tube (Eppendorf, Hamburg, Germany) was mounted in the equatorial plain of the apple fruit using nontoxic silicone rubber (Silicone RTV; Dow Toray, Japan). After curing (approximately 1 h), the tubes were filled with 1 ml deionized water for moisture exposure (Phase I) through a hole in the tip. The hole was then sealed, and the tube was checked for leakage on a daily basis. The opposite side of the fruit served as a control and remained dry [13, 14, 21]. The fruit skin was exposed to moisture (‘Moisture’) for 12 d (‘12 d wet + 0 dry’, (‘Phase I + Phase II’)) during Phase I. For termination of moisture exposure, the tube was removed, and the treated skin patch was exposed to the atmosphere (Phase II). At this point, the treatments were terminated. During the subsequent Phase II, changes in the treated fruit skin patches were observed for up to 136 d (‘12 d wet + 136 d dry’) after termination of the moisture treatment (Phase II).

For wounding-induced periderms, the fruit skin was gently abraded in the equatorial plane using sandpaper (grit size 1000; Bauhaus, Mannheim, Germany) (‘Wounding’). The opposite surface of the same fruit served as the control. Wounding was performed at 38–40 DAFB (Table S3). This time point corresponded to the time of moisture termination in the moisture-induced russeting experiment.

### RNA extraction and quality assessment

Patches of treated, i.e., moisture-exposed or wounded, or nontreated, i.e., control, skins were excised using a razorblade, immediately frozen in liquid N_2_ and held at -80 °C until further analysis. Each replicate comprised skin patches of a minimum of six fruits (approximately 60–80 mg). The tissue was ground in liquid N_2_ to a fine powder using a mortar and pestle. Total RNA was extracted using the InviTrap Spin Plant RNA Mini Kit (STRATEC Molecular GmbH, Berlin, Germany) and lysis buffer RP according to the manufacturer’s protocol. Total RNA was treated with DNase using the DNA-free™ Kit (Thermo Fisher Scientific, Waltham, Massachusetts, USA) to remove remaining DNA. The quantity and purity of RNA were determined photometrically at 230, 260 and 280 nm on a Nanodrop 2000c spectrophotometer (Thermo Fisher Scientific, Waltham, Massachusetts, USA). The integrity and purity of the RNA were checked on a 1.5% agarose gel. Before RNA-Seq, the RNA integrity number (RIN) was determined using the Agilent RNA 6000 Nano Kit on a Bioanalyzer 2100 and the Plant RNA Nano parameters (Agilent Technologies, Santa Clara, CA, USA). The RIN ranged from 8.4 to 10.0 (Table S1).

### RNA-Seq library preparation and sequencing

For each replicate, 1 µg of total RNA was sequenced (Novogene, Cambridge, UK). The library was prepared with the NEBNext® Ultra™ RNA Library Prep Kit (Ipswich, Massachusetts, USA) according to the manufacturer’s instructions. For sequencing, 2 × 150 bp *paired-end* cDNA libraries were prepared. Sequencing was performed on an Illumina® NovaSeq™ 6000. A minimum of 59.8 million read pairs were generated for each sample (Table S1).

### Mapping and counting of reads

Reads obtained from Illumina sequencing were trimmed and filtered with Trimmomatic (v0.39) [80] with the following parameters: TRAILING: 20 AVQUAL: 20 SLIDINGWINDOW: 5:20 MINLEN: 75. The quality of trimmed reads was checked by FastQC (v0.11.9) [81]. Afterward, reads were aligned to the *Malus x domestica HFTH1* v1.0 genome using STAR (v2.5.4b) followed by read count quantification with the “--quantMode Gene Counts” function [82, 83]. Annotations of transcripts were obtained by blastp against the *Arabidopsis thaliana* genome (TAIR10, www.arabidopsis.org, 31.01.2023) as described by Zhang and coworkers [83].

### Differential gene expression and enrichment analysis

Differential gene expression analysis was conducted with DESeq2 (v1.32.0) [84]. Genes with a log_2_FC ≥ 2, ≤ -2 (‘Moisture’ vs. ‘Control’) and a false discovery rate (FDR) ≤ 0.05 were considered to be differentially expressed and used for downstream analysis. Gene abundance was obtained through transcripts per million (TPM) calculation with StringTie (v2.1.3) [85]. Singular enrichment analysis (SEA) was performed with DEGs having a mean of at least five TPM for ’Moisture’ or ‘Control’ samples. Orthologous genes from *Arabidopsis thaliana* were investigated using the webtool AgriGO (v2.0) and the parameters selected species: *Arabidopsis thaliana*; reference: TAIR genome locus (TAIR10_2017), user defined; statistical test method: hypergeometric; multitest adjustment method: Hochberg (FDR); significance level: 0.01; and minimum number of mapping entries: 5 [86]. Heatmaps of differentially expressed genes were generated with the R package ‘pheatmap’ (1.0.12) [87].

### Quantitative real-time PCR

Quantitative real-time PCR (qPCR) was conducted on a QuantStudio™ 6 Flex Real-Time PCR System (Applied Biosystems, Waltham, MA, USA). Primer design, cDNA synthesis, primer efficiency testing and qPCR were performed as described earlier [21] (Table S5). Gene expression values were determined according to Pfaffl [88] with slight modifications described by Chen and coworkers [89]. Gene expression data were normalized using *PROTEIN DISULFIDE ISOMERASE* (PDI) (MDP0000233444) [90] and *MdeF-1alpha* (AJ223969.1) [26] as reference genes. Each data point comprised three independent replicates of two to three technical replicates each.

### Electronic supplementary material

Below is the link to the electronic supplementary material.

Supplementary Material 1

Supplementary Material 2

Supplementary Material 3

Supplementary Material 4

Supplementary Material 5

Supplementary Material 6

Supplementary Material 7

Supplementary Material 8

Supplementary Material 9

Supplementary Material 10

Supplementary Material 11

Supplementary Material 12

Supplementary Material 13

Supplementary Material 14

Supplementary Material 15

Supplementary Material 16

Supplementary Material 17

Supplementary Material 18

Supplementary Material 19

Supplementary Material 20

Supplementary Material 21

Supplementary Material 22

Supplementary Material 23

Supplementary Material 24

## Data Availability

The datasets supporting the conclusions of this article are included within the article and its additional files. Raw Illumina sequencing data are available at the NCBI Sequence Read Archive (SRA) under the BioProject number PRJNA935373.

## Declarations

### Links for review

SRA BioProject (publicly available upon publication): https://dataview.ncbi.nlm.nih.gov/object/PRJNA935373?reviewer=efbmo3ejavqu9s1aeom137tpni.

### Data access for review:

https://my.hidrive.com/share/wymikjrtwa. Password: Russeting.

## List of abbreviations

ABA: Abscisic acid
AP2B3: *AP2/B3-like transcription factor family protein*
AtNCED3: 9-cis-epoxycarotenoid dioxygenase 3
C: Cuticle
DAFB: Days after full bloom
DEGs: Differentially expressed genes
E: Epidermis
EXP: Expansins
FDR: False discovery rate
GDSL: Esterases/lipases
GO: Gene ontology
H: Hypodermis
LAC7: Laccase 7
LEA: *Late embryogenesis abundant hydroxyproline-rich glycoprotein*
log_2_FC: Log_2_-fold change
MC: Microcrack
MYB102: *MYB-like 102*
PCA: Principal component analysis
PD: Phelloderm
PDI: *Protein disulfide isomerase*
PG: Phellogen
PM: Phellem
PRX: Peroxidases
qPCR: Quantitative real-time PCR
QTLs: Quantitative trait loci
SEA: Singular enrichment analysis
SGNH: *SGNH hydrolase*
SM: Surface moisture
TPM: Transcripts per million
>WOX4: *Wuschel-related homeobox 4*
XTH: Xyloglucan endotransglucosylases/hydrolases

## References

[1] Skene DS (1982). The development of russet, rough russet and cracks on the fruit of the apple Cox’s Orange Pippin during the course of the season. J Hortic Sci.

[2] Winkler A, Knoche M, Athoo TO (2020). Russeting in ‘Apple’ mango: triggers and mechanisms. Plants.

[3] Goffinet MC, Pearson RC (1991). Anatomy of russeting induced in Concord grape berries by the fungicide chlorothalonil. Am J Enol Vitic.

[4] Michailides TJ (1991). Russeting and russet scab of prune, an environmentally induced fruit disorder: Symptomatology, induction, and control. Plant Dis.

[5] Scharwies JD, Grimm E, Knoche M. Russeting and relative growth rate are positively related in ‘Conference’ and ‘Condo’ pear. HortScience. 2014;49:746–9.

[6] Lara I, Belge B, Goulao LF (2014). The fruit cuticle as a modulator of postharvest quality. Postharvest Biol Technol.

[7] Lara I, Heredia A, Domínguez E (2019). Shelf life potential and the fruit cuticle: the unexpected player. Front. Plant Sci.

[8] Khanal BP, Ikigu GM, Knoche M (2019). Russeting partially restores apple skin permeability to water vapour. Planta.

[9] Bell HP (1937). The origin of russeting in Golden Russet apple. Can J Res.

[10] Meyer A (1944). A study of the skin structure of Golden Delicious apples. Proc Am Soc Hortic Sci.

[11] Pratt C (1972). Periderm development and radiation stability of russet-fruited sports of apple. Hortic Res.

[12] Faust M, Shear CB (1972). Russeting of apples, an interpretive review. HortScience.

[13] Chen Y-H, Straube J, Khanal BP, Knoche M, Debener T (2020). Russeting in apple is initiated after exposure to moisture ends-I. histological evidence. Plants.

[14] Khanal BP, Imoro Y, Chen YH, Straube J, Knoche M (2020). Surface moisture increases microcracking and water vapour permeance of apple fruit skin. Plant Biol.

[15] Faust M, Shear CB (1972). Fine structure of the fruit surface of three apple cultivars. J Amer Soc Hort Sci.

[16] Khanal BP, Knoche M, Bußler S, Schlüter O (2014). Evidence for a radial strain gradient in apple fruit cuticles. Planta.

[17] Lai X, Khanal BP, Knoche M (2016). Mismatch between cuticle deposition and area expansion in fruit skins allows potentially catastrophic buildup of elastic strain. Planta.

[18] Tukey LD (1959). Observations on the russeting of apples growing in plastic bags. Proc Am Soc Hortic Sci.

[19] Knoche M, Grimm E (2008). Surface moisture induces microcracks in the cuticle of ‘Golden Delicious’ apple. HortScience.

[20] Winkler A, Grimm E, Knoche M, Lindstaedt J, Köpcke D (2014). Late-season surface water induces skin spot in apple. HortScience.

[21] Straube J, Chen Y-H, Khanal BP, Shumbusho A, Zeisler-Diehl V, Suresh K (2020). Russeting in apple is initiated after exposure to moisture ends: Molecular and biochemical evidence. Plants.

[22] Khanal BP, Le TL, Si Y, Knoche M (2020). Russet susceptibility in apple is associated with skin cells that are larger, more variable in size, and of reduced fracture strain. Plants.

[23] Falginella L, Cipriani G, Monte C, Gregori R, Testolin R, Velasco R (2015). A major QTL controlling apple skin russeting maps on the linkage group 12 of ‘Renetta Grigia di Torriana’. BMC Plant Biol.

[24] Lashbrooke J, Aharoni A, Costa F (2015). Genome investigation suggests *MdSHN3*, an APETALA2-domain transcription factor gene, to be a positive regulator of apple fruit cuticle formation and an inhibitor of russet development. J Exp Bot.

[25] Falginella L, Andre CM, Legay S, Lin-Wang K, Dare AP, Deng C (2021). Differential regulation of triterpene biosynthesis induced by an early failure in cuticle formation in apple. Hortic Res.

[26] Legay S, Guerriero G, Deleruelle A, Lateur M, Evers D, André CM, Hausman J-F (2015). Apple russeting as seen through the RNA-seq lens: strong alterations in the exocarp cell wall. Plant Mol Biol.

[27] Legay S, Guerriero G, André C, Guignard C, Cocco E, Charton S (2016). MdMyb93 is a regulator of suberin deposition in russeted apple fruit skins. New Phytol.

[28] Xu X, Guerriero G, Berni R, Sergeant K, Guignard C, Lenouvel A (2022). MdMYB52 regulates lignin biosynthesis upon the suberization process in apple. Front Plant Sci.

[29] Lashbrooke J, Cohen H, Levy-Samocha D, Tzfadia O, Panizel I, Zeisler V (2016). MYB107 and MYB9 homologs regulate suberin deposition in Angiosperms. Plant Cell.

[30] André CM, Guerriero G, Lateur M, Charton S, Leclercq CC, Renaut J (2022). Identification of novel candidate genes involved in apple cuticle integrity and russeting-associated triterpene synthesis using metabolomic, proteomic, and transcriptomic data. Plants.

[31] Yuan G, Bian S, Han X, He S, Liu K, Zhang C, Cong P (2019). An integrated transcriptome and proteome analysis reveals new insights into russeting of bagging and non-bagging Golden Delicious apple. Int J Mol Sci.

[32] Wang Z, Liu S, Huo W, Chen M, Zhang Y, Jiang S (2022). Transcriptome and metabolome analyses reveal phenotype formation differences between russet and non-russet apples. Front Plant Sci.

[33] Taylor BK (1975). Reduction of apple skin russeting by gibberellin. J Hortic Sci.

[34] Taylor BK (1978). Effects of gibberellin sprays on fruit russet and tree performance of Golden Delicious apple. J Hortic Sci.

[35] Wertheim SJ (1982). Fruit russeting in apple as affected by various gibberellins. J Hortic Sci.

[36] Simons RK, Chu MC (1978). Periderm morphology of mature ‘Golden Delicious’ apple with special reference to russeting. Sci Hortic.

[37] Aharoni A, Dixit S, Jetter R, Thoenes E, van Arkel G, Pereira A (2004). The SHINE clade of AP2 domain transcription factors activates wax biosynthesis, alters cuticle properties, and confers drought tolerance when overexpressed in Arabidopsis. Plant Cell.

[38] Shi JX, Malitsky S, de Oliveira S, Branigan C, Franke RB, Schreiber L, Aharoni A (2011). SHINE transcription factors act redundantly to pattern the archetypal surface of Arabidopsis flower organs. PLoS Genet.

[39] Shi JX, Adato A, Alkan N, He Y, Lashbrooke J, Matas AJ (2013). The tomato SlSHINE3 transcription factor regulates fruit cuticle formation and epidermal patterning. New Phytol.

[40] Lee SB, Suh MC (2015). Cuticular wax biosynthesis is up-regulated by the MYB94 transcription factor in Arabidopsis. Plant Cell Physiol.

[41] Lee SB, Kim HU, Suh MC (2016). MYB94 and MYB96 additively activate cuticular wax biosynthesis in Arabidopsis. Plant Cell Physiol.

[42] Liberman LM, Sparks EE, Moreno-Risueno MA, Petricka JJ, Benfey PN. MYB36 regulates the transition from proliferation to differentiation in the Arabidopsis root. Proc. Natl. Acad. Sci. U.S.A. 2015;112:12099–104. 10.1073/pnas.1515576112. 10.1073/pnas.1515576112 PMC4593085 26371322

[43] Chen Y-H, Straube J, Khanal BP, Zeisler-Diehl V, Suresh K, Schreiber L (2022). Apple fruit periderms (russeting) induced by wounding or by moisture have the same histologies, chemistries and gene expressions. PLoS ONE.

[44] Khanal BP, Shrestha R, Hückstädt L, Knoche M (2013). Russeting in apple seems unrelated to the mechanical properties of the cuticle at maturity. HortScience.

[45] Daines R (1984). Effect of early sprays on control of powdery mildew fruit russet on apples. Plant Dis.

[46] Heidenreich MCM, Corral-Garcia MR, Momol EA, Burr TJ (1997). Russet of apple fruit caused by *Aureobasidium pullulans* and Rhodotorula glutinis. Plant Dis.

[47] Gildemacher P, Heijne B, Silvestri M, Houbraken J, Hoekstra E, Theelen B, Boekhout T (2006). Interactions between yeasts, fungicides and apple fruit russeting. FEMS Yeast Res.

[48] Gildemacher PR, Heijne B, Houbraken J, Vromans T, Hoekstra ES, Boekhout T (2004). Can phyllosphere yeasts explain the effect of scab fungicides on russeting of Elstar apples? Eur. J Plant Pathol.

[49] Li C, Yaegashi H, Kishigami R, Kawakubo A, Yamagishi N, Ito T, Yoshikawa N (2020). Apple russet ring and apple green crinkle diseases: fulfillment of Koch’s postulates by virome analysis, amplification of full-length cDNA of viral genomes, *in vitro* transcription of infectious viral RNAs, and reproduction of symptoms on fruits of apple trees inoculated with viral RNAs. Front Microbiol.

[50] Wood GA (1972). Russet ring and some associated virus disorders of apple (*Malus sylvestris* (L.) Mill.) In New Zealand. New Z J Agric Res.

[51] Welsh MF, May J (1967). Virus etiology of foliar vein-flecking or ring pattern and fruit russeting or blotch on apple. Can J Plant Sci.

[52] Easterbrook MA, Fuller MM (1986). Russeting of apples caused by apple rust mite *Aculus schlechtendali* (Acarina: Eriophyidae). Ann Appl Biol.

[53] Winkler A, Athoo T, Knoche M (2022). Russeting of fruits: etiology and management. Horticulturae.

[54] Creasy LL (1980). The correlation of weather parameters with russet of Golden Delicious apples under orchard conditions. J Amer Soc Hort Sci.

[55] Petit J, Bres C, Mauxion JP, Tai F, Martin LB, Fich EA et al. The glycerol-3-phosphate acyltransferase GPAT6 from tomato plays a central role in fruit cutin biosynthesis. Plant Physiol. 2016:894–913. 10.1104/pp.16.00409 PMC4902622 27208295

[56] Li Y, Beisson F, Koo AJK, Molina I, Pollard M, Ohlrogge J. Identification of acyltransferases required for cutin biosynthesis and production of cutin with suberin-like monomers. Proc. Natl. Acad. Sci. U.S.A. 2007;104:18339–44. 10.1073/pnas.0706984104. 10.1073/pnas.0706984104 PMC2084344 17991776

[57] Bird D, Beisson F, Brigham A, Shin J, Greer S, Jetter R, et al. Characterization of Arabidopsis ABCG11/WBC11, an ATP binding cassette (ABC) transporter that is required for cuticular lipid secretion. Plant J. 2007;485–98. 10.1111/j.1365-313X.2007.03252.x. 10.1111/j.1365-313X.2007.03252.x 17727615

[58] Jakoby MJ, Falkenhan D, Mader MT, Brininstool G, Wischnitzki E, Platz N (2008). Transcriptional profiling of mature Arabidopsis trichomes reveals that *NOECK* encodes the MIXTA-like transcriptional regulator MYB106. Plant Physiol.

[59] Oshima Y, Mitsuda N (2013). The MIXTA-like transcription factor MYB16 is a major regulator of cuticle formation in vegetative organs. Plant Signal Behav.

[60] Xu B, Sathitsuksanoh N, Tang Y, Udvardi MK, Zhang J-Y, Shen Z (2012). Overexpression of *AtLOV1* in Switchgrass alters plant architecture, lignin content, and flowering time. PLoS ONE.

[61] Khanal BP, Knoche M (2017). Mechanical properties of cuticles and their primary determinants. J Exp Bot.

[62] Khanal BP, Knoche M (2014). Mechanical properties of apple skin are determined by epidermis and hypodermis. J Amer Soc Hort Sci.

[63] Knoche M, Khanal BP, Brüggenwirth M, Thapa S (2018). Patterns of microcracking in apple fruit skin reflect those of the cuticular ridges and of the epidermal cell walls. Planta.

[64] Xiao W, Molina D, Wunderling A, Ripper D, Vermeer JEM, Ragni L (2020). Pluripotent pericycle cells trigger different growth outputs by integrating developmental cues into distinct regulatory modules. Curr Biol.

[65] Wunderling A, Ripper D, Barra-Jimenez A, Mahn S, Sajak K, Targem MB, Ragni L (2018). A molecular framework to study periderm formation in Arabidopsis. New Phytol.

[66] Almeida T, Pinto G, Correia B, Santos C, Gonçalves S (2013). *QsMYB1* expression is modulated in response to heat and drought stresses and during plant recovery in *Quercus suber*. Plant Physiol Biochem.

[67] Almeida T, Menéndez E, Capote T, Ribeiro T, Santos C, Gonçalves S (2013). Molecular characterization of *Quercus suber MYB1*, a transcription factor up-regulated in cork tissues. J Plant Physiol.

[68] Leal AR, Barros PM, Parizot B, Sapeta H, Vangheluwe N, Andersen TG (2022). Translational profile of developing phellem cells in *Arabidopsis thaliana* roots. Plant J.

[69] Ji J, Strable J, Shimizu R, Koenig D, Sinha N, Scanlon MJ (2010). WOX4 promotes procambial development. Plant Physiol.

[70] Hirakawa Y, Kondo Y, Fukuda H (2010). TDIF peptide signaling regulates vascular stem cell proliferation via the *WOX4* homeobox gene in Arabidopsis. Plant Cell.

[71] Etchells JP, Provost CM, Mishra L, Turner SR (2013). WOX4 and WOX14 act downstream of the PXY receptor kinase to regulate plant vascular proliferation independently of any role in vascular organisation. Development.

[72] Zhang J, Eswaran G, Alonso-Serra J, Kucukoglu M, Xiang J, Yang W (2019). Transcriptional regulatory framework for vascular cambium development in Arabidopsis roots. Nat Plants.

[73] Kucukoglu M, Nilsson J, Zheng B, Chaabouni S, Nilsson O (2017). *Wuschel-related homeobox4* (*WOX4*)-like genes regulate cambial cell division activity and secondary growth in Populus trees. New Phytol.

[74] Sato H, Takasaki H, Takahashi F, Suzuki T, Iuchi S, Mitsuda N (2018). Arabidopsis thaliana NGATHA1 transcription factor induces ABA biosynthesis by activating NCED3 gene during dehydration stress. Proc Natl Acad Sci U S A.

[75] Battaglia M, Olvera-Carrillo Y, Garciarrubio A, Campos F, Covarrubias AA (2008). The enigmatic LEA proteins and other hydrophilins. Plant Physiol.

[76] Vilaine F, Kerchev P, Clément G, Batailler B, Cayla T, Bill L (2013). Increased expression of a phloem membrane protein encoded by *NHL26* alters phloem export and sugar partitioning in Arabidopsis. Plant Cell.

[77] Barberon M, Vermeer JEM, de Bellis D, Wang P, Naseer S, Andersen TG (2016). Adaptation of root function by nutrient-induced plasticity of endodermal differentiation. Cell.

[78] Denekamp M, Smeekens SC (2003). Integration of wounding and osmotic stress signals determines the expression of the *AtMYB102* transcription factor gene. Plant Physiol.

[79] Ursache R, de Jesus Vieira Teixeira C, Dénervaud Tendon V, Gully K, de Bellis D, Schmid-Siegert E (2021). GDSL-domain proteins have key roles in suberin polymerization and degradation. Nat Plants.

[80] Bolger AM, Lohse M, Usadel B (2014). Trimmomatic: a flexible trimmer for Illumina sequence data. Bioinformatics.

[81] Andrews S, FastQC:. A Quality Control Tool for High Throughput Sequence Data.; 2010.

[82] Dobin A, Davis CA, Schlesinger F, Drenkow J, Zaleski C, Jha S (2013). STAR: ultrafast universal RNA-seq aligner. Bioinformatics.

[83] Zhang L, Hu J, Han X, Li J, Gao Y, Richards CM (2019). A high-quality apple genome assembly reveals the association of a retrotransposon and red fruit colour. Nat Commun.

[84] Love MI, Huber W, Anders S (2014). Moderated estimation of fold change and dispersion for RNA-seq data with DESeq2. Genome Biol.

[85] Pertea M, Pertea GM, Antonescu CM, Chang T-C, Mendell JT, Salzberg SL (2015). StringTie enables improved reconstruction of a transcriptome from RNA-seq reads. Nat Biotechnol.

[86] Tian T, Liu Y, Yan H, You Q, Yi X, Du Z (2017). agriGO v2.0: a GO analysis toolkit for the agricultural community, 2017 update. Nucleic Acids Res.

[87] Kolde R. Pheatmap: pretty heatmap; 2019.

[88] Pfaffl MW (2001). A new mathematical model for relative quantification in real-time RT-PCR. Nucleic Acids Res.

[89] Chen Y-H, Khanal BP, Linde M, Debener T, Alkio M, Knoche M (2019). Expression of putative aquaporin genes in sweet cherry is higher in flesh than skin and most are downregulated during development. Sci Hortic.

[90] Storch TT, Pegoraro C, Finatto T, Quecini V, Rombaldi CV, Girardi CL (2015). Identification of a novel reference gene for apple transcriptional profiling under postharvest conditions. PLoS ONE.

