# Supplementary material for: Time course of changes in the transcriptome during russet induction in apple fruit
Source: BMC Plant Biol. 2023 Sep 30;23:457. doi: 10.1186/s12870-023-04483-6 (PMC10542230; doi:10.1186/s12870-023-04483-6)
Supplement: Supplementary file 1 — Supplementary Material 1 [file 12870_2023_4483_MOESM1_ESM.docx]

**Table S2.** Summary of differentially expressed genes detected in the RNA-seq analysis. Genes with a log_2-_fold change (log_2_FC) ≥ 2 or ≤ -2, a false discovery rate (FDR) ≤ 0.05 and a mean of at least five TPM (transcripts per million) in control (‘x d dry + y d dry’) or moisture-exposed (‘x d wet + y d dry’) samples were assumed to be differentially expressed.

| Season | Time point | Number up-regulated genes  (log_2_FC ≥ 2, FDR ≤ 0.05) | Number down-regulated genes  (log_2_FC ≤ -2, FDR ≤ 0.05) |
| --- | --- | --- | --- |
|  |  |  |  |
| **2018** | ‘0 d wet + 0 d dry’ vs ‘0 d dry + 0 d dry’ | 0 | 0 |
|  | ‘2 d wet + 0 d dry’ vs ‘2 d dry+ 0 d dry’ | 35 | 2 |
|  | ‘6 d wet + 0 d dry’ vs ‘6 d dry+ 0 d dry’ | 378 | 127 |
|  | ‘12 d wet + 0 d dry’ vs ‘12 d dry+ 0 d dry’ | 860 | 196 |
|  | ‘12 d wet + 8 d dry’ vs ‘12 d dry + 8 d dry’ | 523 | 44 |
| **2019** | ‘0 d wet + 0 d dry’ vs ‘0 d dry+ 0 d dry’ | 0 | 0 |
|  | ‘12 d wet + 0 d dry’ vs ‘12 d dry+ 0 d dry’ | 1219 | 858 |
|  | ‘12 d wet + 1 d dry’ vs ‘12 d dry + 1 d dry’ | 1094 | 597 |
|  | ‘12 d wet + 3 d dry’ vs ‘12 d dry + 3 d dry’ | 980 | 321 |
|  | ‘12 d wet + 8 d dry’ vs ‘12 d dry + 8 d dry’ | 974 | 253 |
|  |  |  |  |
