# Supplementary material for: Time course of changes in the transcriptome during russet induction in apple fruit
Source: BMC Plant Biol. 2023 Sep 30;23:457. doi: 10.1186/s12870-023-04483-6 (PMC10542230; doi:10.1186/s12870-023-04483-6)
Supplement: Supplementary file 2 — Supplementary Material 2 [file 12870_2023_4483_MOESM2_ESM.docx]

**Table S1.** Summary of RNA integrity number (RIN), sequencing results, trimming and uniquely mapped reads of the HFTH1 genome.

| Season | Time point | | Biological replicate | | RIN | Raw reads | Read pairs after trimming (Q ≥ 20) | Percentage of uniquely mapped reads (%) |
| --- | --- | --- | --- | --- | --- | --- | --- | --- |
|  |  | |  | |  |  |  |  |
| **2018** | ‘0 d dry + 0 d dry’ | | 1 | | 8.8 | 121640060 | 57643670 | 94.25 |
|  | ‘0 d dry + 0 d dry’ | | 2 | | 9.4 | 128454744 | 61140644 | 95.18 |
|  | ‘0 d dry + 0 d dry’ | | 3 | | 9.1 | 123418270 | 57636005 | 94.24 |
|  | ‘0 d wet + 0 d dry’ | | 1 | | 9.8 | 150068214 | 70456595 | 93.88 |
|  | ‘0 d wet + 0 d dry’ | | 2 | | 9.4 | 150250946 | 70539939 | 94.72 |
|  | ‘0 d wet + 0 d dry’ | | 3 | | 9.2 | 141769224 | 66661960 | 94.51 |
|  | ‘2 d dry + 0 d dry’ | | 1 | | 9.9 | 123879264 | 58832415 | 94.45 |
|  | ‘2 d dry + 0 d dry’ | | 2 | | 9.6 | 128791720 | 61108589 | 94.22 |
|  | ‘2 d dry + 0 d dry’ | | 3 | | 9.8 | 124941598 | 58925324 | 93.99 |
|  | ‘2 d wet + 0 d dry’ | | 1 | | 9.6 | 130739500 | 62363018 | 94.60 |
|  | ‘2 d wet + 0 d dry’ | | 2 | | 9.7 | 121474696 | 57692471 | 94.34 |
|  | ‘2 d wet + 0 d dry’ | | 3 | | 8.4 | 123936980 | 58570379 | 93.34 |
|  | ‘6 d dry + 0 d dry’ | | 1 | | 9.3 | 122418652 | 58284983 | 94.95 |
|  | ‘6 d dry + 0 d dry’ | | 2 | | 9.8 | 120848968 | 57802019 | 93.86 |
|  | ‘6 d dry + 0 d dry’ | | 3 | | 9.9 | 124544584 | 59079988 | 95.11 |
|  | ‘6 d wet + 0 d dry’ | | 1 | | 9.8 | 123095348 | 58342147 | 90.33 |
|  | ‘6 d wet + 0 d dry’ | | 2 | | 9.6 | 126869850 | 60166854 | 82.89 |
|  | ‘6 d wet + 0 d dry’ | | 3 | | 9.8 | 127060892 | 60185343 | 90.32 |
|  | ‘12 d dry + 0 d dry’ | | 1 | | 8.8 | 119799220 | 56896654 | 93.84 |
|  | ‘12 d dry + 0 d dry’ | | 2 | | 9.3 | 128534002 | 60734209 | 93.79 |
|  | ‘12 d dry + 0 d dry’ | | 3 | | 8.8 | 123421432 | 58359708 | 93.56 |
|  | ‘12 d wet + 0 d dry’ | | 1 | | 9.9 | 123129710 | 58346280 | 92.98 |
|  | ‘12 d wet + 0 d dry’ | | 2 | | 10 | 122313942 | 58141580 | 93.17 |
|  | ‘12 d wet + 0 d dry’ | | 3 | | 9.7 | 123117832 | 58488243 | 93.14 |
|  | ‘12 d dry + 8 d dry’ | | 1 | | 9.3 | 121948016 | 57759425 | 94.77 |
|  | ‘12 d dry + 8 d dry’ | | 2 | | 10 | 121920638 | 57687317 | 95.40 |
|  | ‘12 d dry + 8 d dry’ | | 3 | | 10 | 124020262 | 58715966 | 95.52 |
|  | ‘12 d wet + 8 d dry’ | | 1 | | 8.8 | 122072708 | 57970170 | 94.28 |
|  | ‘12 d wet + 8 d dry’ | | 2 | | 9.9 | 124268364 | 58801142 | 94.45 |
|  | ‘12 d wet + 8 d dry’ | | 3 | | 9.9 | 146351032 | 69089407 | 94.46 |
| **2019** | ‘0 d dry + 0 d dry’ | | 1 | | 9.7 | 133889700 | 63261870 | 94.88 |
|  | ‘0 d dry + 0 d dry’ | | 2 | | 9.6 | 119689572 | 56591648 | 94.14 |
|  | ‘0 d dry + 0 d dry’ | | 3 | | 10 | 125224450 | 59312334 | 93.81 |
|  | ‘0 d wet + 0 d dry’ | | 1 | | 9.9 | 137475904 | 64990547 | 94.55 |
|  | ‘0 d wet + 0 d dry’ | | 2 | | 9.7 | 124077252 | 57751485 | 93.64 |
|  | ‘0 d wet + 0 d dry’ | | 3 | | 9.8 | 122223580 | 57277739 | 93.63 |
|  | ‘12 d dry + 0 d dry’ | | 1 | | 9.5 | 121552076 | 57900489 | 94.52 |
|  | ‘12 d dry + 0 d dry’ | | 2 | | 9.8 | 130935978 | 61992895 | 94.02 |
|  | ‘12 d dry + 0 d dry’ | | 3 | | 9.7 | 129075842 | 61201544 | 94.55 |
|  | ‘12 d wet + 0 d dry’ | | 1 | | 9.7 | 123881026 | 58419444 | 93.29 |
|  | ‘12 d wet + 0 d dry’ | | 2 | | 10 | 122233530 | 57745233 | 94.04 |
|  | ‘12 d wet + 0 d dry’ | | 3 | | 9.9 | 126287244 | 59665259 | 93.90 |
|  | ‘12 d dry + 1 d dry’ | | 1 | | 9.7 | 128612160 | 60984921 | 94.67 |
|  | ‘12 d dry + 1 d dry’ | | 2 | | 9.8 | 123635836 | 59317591 | 93.80 |
|  | ‘12 d dry + 1 d dry’ | | 3 | | 9.6 | 123068870 | 57441459 | 93.88 |
|  | ‘12 d wet + 1 d dry’ | | 1 | | 10 | 122987230 | 57351930 | 94.11 |
|  | ‘12 d wet + 1 d dry’ | | 2 | | 10 | 121859564 | 57339366 | 93.74 |
|  | ‘12 d wet + 1 d dry’ | | 3 | | 10 | 119907316 | 56615752 | 93.94 |
|  | ‘12 d dry + 3 d dry’ | | 1 | | 9.5 | 123202362 | 58500246 | 94.47 |
|  | ‘12 d dry + 3 d dry’ | | 2 | | 9.8 | 122097110 | 57908035 | 94.03 |
|  | ‘12 d dry + 3 d dry’ | | 3 | | 9.6 | 122280412 | 57978514 | 94.02 |
|  | ‘12 d wet + 3 d dry’ | | 1 | | 10 | 121481146 | 57128431 | 94.79 |
|  | ‘12 d wet + 3 d dry’ | | 2 | | 9.8 | 133529112 | 63057053 | 94.68 |
|  | ‘12 d wet + 3 d dry’ | | 3 | | 10 | 123702882 | 58426916 | 94.46 |
|  | ‘12 d dry + 8 d dry’ | | 1 | | 9.8 | 132175454 | 62733354 | 94.62 |
|  | ‘12 d dry + 8 d dry’ | | 2 | | 9.9 | 123778558 | 58738285 | 94.37 |
|  | ‘12 d dry + 8 d dry’ | | 3 | | 9.8 | 121345836 | 56774298 | 94.04 |
|  | ‘12 d wet + 8 d dry’ | | 1 | | 10 | 123826376 | 58422565 | 95.22 |
|  | ‘12 d wet + 8 d dry’ | | 2 | | 10 | 124522250 | 58469468 | 94.45 |
|  | ‘12 d wet + 8 d dry’ | | 3 | | 10 | 123605066 | 58066909 | 94.74 |
|  |  |  | |  | |  |  |  |
|  | | | | | | | | |
