# Supplementary material for: Time course of changes in the transcriptome during russet induction in apple fruit
Source: BMC Plant Biol. 2023 Sep 30;23:457. doi: 10.1186/s12870-023-04483-6 (PMC10542230; doi:10.1186/s12870-023-04483-6)
Supplement: Supplementary file 3 — Supplementary Material 3 [file 12870_2023_4483_MOESM3_ESM.docx]

**Table S4.** Russeting after mechanical wounding during early fruit development (38-40 days after full bloom (DAFB)) of ‘Karmijn’, ‘Pinova’, ‘Idared’ and ‘Gala’ apples in the 2022 season. Russeting was quantified at commercial maturity.

| Cultivar | Developmental stage (DAFB) | Number of fruits | Frequency of russeted fruits (%) | Russeted area  (% of treated area) |
| --- | --- | --- | --- | --- |
|  |  |  |  | Wounded |
| ‘Karmijn’ | 159 | 35 | 100 | 100 |
| ‘Pinova’ | 145 | 49 | 100 | 100 |
| ‘Idared’ | 173 | 46 | 100 | 100 |
| ‘Gala’ | 146 | 49 | 100 | 100 |
|  |  |  |  |  |
