# Supplementary material for: Time course of changes in the transcriptome during russet induction in apple fruit
Source: BMC Plant Biol. 2023 Sep 30;23:457. doi: 10.1186/s12870-023-04483-6 (PMC10542230; doi:10.1186/s12870-023-04483-6)
Supplement: Supplementary file 8 — Supplementary Material 8 [file 12870_2023_4483_MOESM8_ESM.docx]

**Table S5.** List of primers used in this study.

|  | | | | | | | | | |  |
| --- | --- | --- | --- | --- | --- | --- | --- | --- | --- | --- |
|  |  | | **Primer sequence (5'-3')** | | |  | |  | |  |
| **Gene name** | | **Accession** | | **Forward Primer** | **Reverse Primer** | | **PCR efficiency (%)** | | **Reference** | |
| **Phase I related** | |  | |  |  | |  | |  | |
| *MYB17* | | HF15264 | | CCACAAACACACGAGCCTCT | GCCTAGCACTCTCCCATTGT | | 94.2 | | This study | |
| *NAC035* | | HF34490 | | CGGAGTTGAAGACCACCCAT | GGTCGTCGATTGAGCCACTA | | 102.5 | | This study | |
| **Phase II related** | |  | |  |  | |  | |  | |
| *AP2B3* | | HF20086 | | CCACACGGATCGACTCTTCAT | CACCACCACTGCTACTCTCAA | | 85.7 | | This study | |
| *MYB52* | | HF24488 | | CCCAAGAATCAACAGGAACCCT | GCCTTGCAATAACAGCCCATC | | 82.9 | | This study | |
| *MYB67* | | HF11445 | | ACATCTGGGCTTCTGGTACTG | TTCCATCTGATCCAAGAGCGT | | 89.4 | | This study | |
| *MYB84* | | HF13180 | | AGTTCTTCTGCTCCTGTGGC | ATTACCGCCTTGCCCTTGAA | | 90.5 | | This study | |
| *MYB93* | | MDP0000320772 | | TGGACAAACTATCTTAGGCCGG | GTTGCCGAGGATGGAATGGA | | 102.5 | | [1] | |
| *MYB102* | | HF33626 | | ACAAATGGTCAGGTATTGCAGC | CCCATTCGGAGGAGCCTTTT | | 93.6 | | This study | |
| *WOX4* | | HF13050 | | TGGTTCCAGAATCACAAGGCA | CCAGAGGAGTCGGTGTTCTT | | 92.3 | | This study | |
| *WRKY56* | | HF14837 | | ATTAGTGGCTCTGCTGGGAAT | CGAGTCTGGAACGCAAACCT | | 90.8 | | This study | |
| *SGNH* | | HF32136 | | CCCAGGGGCAAAACTCTCAT | TGCAACACGGAAGGTTCGAA | | 97.2 | | This study | |
| *LEA* | | HF26067 | | TGCCACCACAATTCTCTCCA | TGAGTTGGACGGACGAGTTG | | 97.9 | | This study | |
| **Reference genes** | |  | |  |  | |  | |  | |
| *eF-1alpha* | | AJ223969.1 | | ACTGTTCCTGTTGGACGTGTTG | TGGAGTTGGAAGCAACGTACCC | | 93.0 | | [2] | |
| *PDI* | | MDP0000233444 | | TGCTGTACACAGCCAACGAT | CATCTTTAGCGGCGTTATCC | | 100.6 | | [3] | |
|  | |  | |  |  | |  | |  | |

**References**

1. Straube J, Chen Y-H, Khanal BP, Shumbusho A, Zeisler-Diehl V, Suresh K, et al. Russeting in apple is initiated after exposure to moisture ends: Molecular and biochemical evidence. Plants 2020. doi:10.3390/plants10010065.

2. Legay S, Guerriero G, Deleruelle A, Lateur M, Evers D, André CM, Hausman J-F. Apple russeting as seen through the RNA-seq lens: strong alterations in the exocarp cell wall. Plant Mol Biol. 2015;88:21–40. doi:10.1007/s11103-015-0303-4.

3. Storch TT, Pegoraro C, Finatto T, Quecini V, Rombaldi CV, Girardi CL. Identification of a novel reference gene for apple transcriptional profiling under postharvest conditions. PLoS One. 2015;10:e0120599. doi:10.1371/journal.pone.0120599.
