# Supplementary material for: Time course of changes in the transcriptome during russet induction in apple fruit
Source: BMC Plant Biol. 2023 Sep 30;23:457. doi: 10.1186/s12870-023-04483-6 (PMC10542230; doi:10.1186/s12870-023-04483-6)
Supplement: Supplementary file 9 — Supplementary Material 9 [file 12870_2023_4483_MOESM9_ESM.docx]

**Table S3.** Sampling time points in the 2018, 2019, 2020 and 2022 growing seasons for experiments on gene expression and histology of apple fruit skins.

| Season | Time point | | Treatment | Cultivar | DAFB | Date |
| --- | --- | --- | --- | --- | --- | --- |
|  |  | |  |  |  |  |
| **2018** | ‘0 d wet + 0 d dry’ vs ‘0 d dry + 0 d dry’ | | Moisture | ‘Pinova’ | 21 | 2018-05-13 |
|  | ‘2 d + 0 d dry’ wet’ vs ‘2 d dry+ 0 d dry’ | | Moisture | ‘Pinova’ | 23 | 2018-05-15 |
|  | ‘6 d wet + 0 d dry’ vs ‘6 d dry+ 0 d dry’ | | Moisture | ‘Pinova’ | 27 | 2018-05-19 |
|  | ‘12 d wet + 0 d dry’ vs ‘12 d dry+ 0 d dry’ | | Moisture | ‘Pinova’ | 33 | 2018-05-25 |
|  | ‘12 d wet + 8 d dry’ vs ‘12 d dry + 8 d dry’ | | Moisture | ‘Pinova’ | 41 | 2018-06-01 |
|  | ‘12 d wet + 111 d dry’ vs. ‘12 d dry + 111 d dry’ | | Moisture | ‘Pinova’ | 152 | 2018-09-20 |
| **2019** | ‘0 d wet + 0 d dry’ vs ‘0 d dry + 0 d dry’ | | Moisture | ‘Pinova’ | 31 | 2019-05-23 |
|  | ‘8 d wet + 0 d dry’ vs ‘8 d dry + 0 d dry’ | | Moisture | ‘Pinova’ | 39 | 2019-05-31 |
|  | ‘12 d wet + 0 d dry’ vs ‘12 d dry+ 0 d dry’ | | Moisture | ‘Pinova’ | 43 | 2019-06-04 |
|  | ‘12 d wet + 1 d dry’ vs. ‘12 d dry + 1 d dry’ | | Moisture | ‘Pinova’ | 44 | 2019-06-05 |
|  | ‘12 d wet + 2 d dry’ vs. ‘12 d dry + 2 d dry’ | | Moisture | ‘Pinova’ | 45 | 2019-06-06 |
|  | ‘12 d wet + 3 d dry’ vs. ‘12 d dry + 3 d dry’ | | Moisture | ‘Pinova’ | 46 | 2019-06-07 |
|  | ‘12 d wet + 4 d dry’ vs. ‘12 d dry + 4 d dry’ | | Moisture | ‘Pinova’ | 47 | 2019-06-08 |
|  | ‘12 d wet + 8 d dry’ vs ‘12 d dry + 8 d dry’ | | Moisture | ‘Pinova’ | 51 | 2019-06-12 |
|  | ‘12 d wet + 113 d dry’ vs. ‘12 d dry + 113 d dry’ | | Moisture | ‘Pinova’ | 156 | 2019-09-25 |
| **2020** | ‘12 d wet + 0 d dry’ vs ‘12 d dry+ 0 d dry’ | | Moisture | ‘Pinova’ | 40 | 2020-06-02 |
|  | ‘12 d wet + 2 d dry’ vs. ‘12 d dry + 2 d dry’ | | Moisture | ‘Pinova’ | 42 | 2020-06-04 |
|  | ‘12 d wet + 4 d dry’ vs. ‘12 d dry + 4 d dry’ | | Moisture | ‘Pinova’ | 44 | 2020-06-06 |
|  | ‘12 d wet + 8 d dry’ vs ‘12 d dry + 8 d dry’ | | Moisture | ‘Pinova’ | 48 | 2020-06-10 |
|  | ‘12 d wet + 111 d dry’ vs. ‘12 d dry + 111 d dry’ | | Moisture | ‘Pinova’ | 151 | 2020-09-21 |
|  | ‘0 d wounding’ vs. ‘0 d control’ | | Wounding | ‘Pinova’ | 40 | 2020-06-02 |
|  | ‘2 d wounding’ vs. ‘2 d control’ | | Wounding | ‘Pinova’ | 42 | 2020-06-04 |
|  | ‘4 d wounding’ vs. ‘4 d control’ | | Wounding | ‘Pinova’ | 44 | 2020-06-06 |
|  | ‘8 d wounding’ vs. ‘8 d control’ | | Wounding | ‘Pinova’ | 48 | 2020-06-10 |
|  | ‘0 d wet + 0 d dry’ vs ‘0 d dry + 0 d dry’ | | Moisture | ‘Karmijn’ | 28 | 2020-05-23 |
|  | ‘12 d wet+ 0 d dry’ vs ‘12 d dry+ 0 d dry’ | | Moisture | ‘Karmijn’ | 40 | 2020-06-04 |
|  | ‘12 d wet + 8 d dry’ vs ‘12 d dry + 8 d dry’ | | Moisture | ‘Karmijn’ | 48 | 2020-06-12 |
|  | ‘12 d wet + 109 d dry’ vs. ‘12 d dry + 109 d dry’ | | Moisture | ‘Karmijn’ | 149 | 2020-09-21 |
|  | ‘0 d wet + 0 d dry’ vs ‘0 d dry + 0 d dry’ | | Moisture | ‘Pinova’ | 30 | 2020-05-23 |
|  | ‘12 d wet + 0 d dry’ vs ‘12 d dry+ 0 d dry’ | | Moisture | ‘Pinova’ | 42 | 2020-06-04 |
|  | ‘12 d wet + 8 d dry’ vs ‘12 d dry + 8 d dry’ | | Moisture | ‘Pinova’ | 50 | 2020-06-12 |
|  | ‘12 d wet + 109 d dry’ vs. ‘12 d dry + 109 d dry’ | | Moisture | ‘Pinova’ | 151 | 2020-09-21 |
|  | ‘0 d wet + 0 d dry’ vs ‘0 d dry + 0 d dry’ | | Moisture | ‘Idared’ | 32 | 2020-05-23 |
|  | ‘12 d wet + 0 d dry’ vs ‘12 d dry+ 0 d dry’ | | Moisture | ‘Idared’ | 44 | 2020-06-04 |
|  | ‘12 d wet + 8 d dry’ vs ‘12 d dry + 8 d dry’ | | Moisture | ‘Idared’ | 52 | 2020-06-12 |
|  | ‘12 d wet + 136 d dry’ vs. ‘12 d dry + 136 d dry’ | | Moisture | ‘Idared’ | 180 | 2020-10-26 |
|  | ‘0 d wet + 0 d dry’ vs ‘0 d dry + 0 d dry’ | | Moisture | ‘Gala’ | 29 | 2020-05-23 |
|  | ‘12 d wet + 0 d dry’ vs ‘12 d dry+ 0 d dry’ | | Moisture | ‘Gala’ | 41 | 2020-06-04 |
|  | ‘12 d wet + 8 d dry’ vs ‘12 d dry + 8 d dry’ | | Moisture | ‘Gala’ | 49 | 2020-06-12 |
|  | ‘12 d wet + 109 d dry’ vs. ‘12 d dry + 109 d dry’ | | Moisture | ‘Gala’ | 150 | 2020-09-21 |
| **2022** | ‘8 d wounding’ vs. ‘8 d control’ | | Wounding | ‘Karmijn’ | 38 | 2022-06-09 |
|  | ‘119 d wounding’ vs. ‘119 d control’ | | Wounding | ‘Karmijn’ | 159 | 2022-10-06 |
|  | ‘8 d wounding’ vs. ‘8 d control’ | | Wounding | ‘Pinova’ | 39 | 2022-06-09 |
|  | ‘106 d wounding’ vs. ‘106 d control’ | | Wounding | ‘Pinova’ | 145 | 2022-09-23 |
|  | ‘8 d wounding’ vs. ‘8 d control’ | | Wounding | ‘Idared’ | 40 | 2022-06-09 |
|  | ‘133 d wounding’ vs. ‘133 d control’ | | Wounding | ‘Idared’ | 173 | 2022-10-20 |
|  | ‘8 d wounding’ vs. ‘8 d control’ | | Wounding | ‘Gala’ | 40 | 2022-06-09 |
|  | ‘106 d wounding’ vs. ‘106 d control’ | | Wounding | ‘Gala’ | 146 | 2022-09-23 |
|  | |  |  |  |  |  |
