# Supplementary material for: Time course of changes in the transcriptome during russet induction in apple fruit
Source: BMC Plant Biol. 2023 Sep 30;23:457. doi: 10.1186/s12870-023-04483-6 (PMC10542230; doi:10.1186/s12870-023-04483-6)
Supplement: Supplementary file 10 — Supplementary Material 10 [file 12870_2023_4483_MOESM10_ESM.docx]

**Method S1 Cross sectioning and microscopy of fruit skin segments**

Apple fruit skin segments of moisture-exposed and non-exposed patches were preserved in Karnovsky fixative [1] and stored at 4 °C. Embedding of tissue blocks and sectioning was conducted as described in previous studies [2–4]. Tissue blocks were rinsed in deionized water and then immersed in 70% (v/v) aqueous ethanol (EtOH) for 16 h, followed by dehydration in a series of EtOH solutions of increasing concentration (80%, 90%, and 96% EtOH, each for 30 minutes). The tissue blocks were then placed in 100% isopropanol for two cycles of 40 minutes each, followed by incubation in a xylene substitute (AppiClear AppliChem, Münster, Germany) for two cycles of 40 minutes each. Subsequently, the tissue blocks were infiltrated with a mixture of paraffin and xylene substitute in a 1:1 (v/v) ratio (Carl Roth, Karlsruhe, Germany) for 40 minutes, and then with pure paraffin for two cycles of 40 minutes each. All these steps were performed under vacuum conditions (absolute pressure 10.8 kPa). The embedded tissue blocks were stored at 4 °C. To prepare the skin sections, the blocks were cut into 10 µm thick slices using a rotary microtome (Hydrax M 55, Zeiss, Oberkochen, Germany). The slices were then transferred to microscope slides and dried at 37 °C for 16 hours. Prior to staining, paraffin was removed by using a xylene substitute for two cycles of 10 minutes each. Next, the tissue was rehydrated by treating it with a series of decreasing concentrations of ethanol (96%, 80%, 70%, and 60% EtOH, each for 10 minutes). Finally, the sections were transferred to deionized water for two cycles of 5 minutes each. Skin sections were stained with 0.005% Fluorol Yellow 088 (Santa Cruz Biotechnology, TX, USA) [5] dissolved in polyethylene glycol 4000 (SERVA Electrophoresis, Heidelberg, Germany) and 90% glycerol mixture (1:1). Microscopic observations were conducted with a fluorescence microscope (BX-60; Olympus, Hamburg, Germany) under transmitted white light as well as incident fluorescent light (filter U-MWB, 450-480 nm excitation; ≥ 520 nm emission wavelength).

**Method S2 Observations on microcrack formation**

Quantification of microcracks in ‘Karmijn’, ‘Pinova’, ‘Idared’ and ‘Gala’ after moisture exposure for 12 d during the 2020 growing season was done as previously described [2, 4, 6, 7]. Briefly, a fruit was immersed in 0.1% (w/v) aqueous acridine orange (Carl Roth, Karlsruhe, Germany) for 10 min. The fruit was then rinsed with deionized water and gently dried with soft tissue paper. The moisture-exposed areas and the nontreated control areas were observed under a fluorescence microscope (MZ10F; GFP-plus filter, 440-480 nm excitation wavelength, ≥ 510 nm emission wavelength; Leica Microsystems, Wetzlar, Germany). Three to four images (DP71; Olympus Europa, Hamburg, Germany) were taken on different areas of the moisture exposed as well as on control fruit skin patches of a total of ten fruits per cultivar each time point. Acridine orange infiltrated areas (mm^2^) were quantified using the image analysis software Cell^P^ (Olympus, Hamburg, Germany) and presented as percentage of infiltrated area.

**Method S3 Evaluation of russet quantity at maturity**

After discontinuing the moisture exposure, the treated area was carefully labeled with a black permanent marker. Photographs were taken from treated fruit skin patches with a Canon EOS 550D camera (lens: EF-S 18-55 mm, Canon Germany, Krefeld, Germany) at maturity. Images were digitally calibrated and areas (mm^2^) showing russeting within the treated fruit skin patches quantified (cellSens; Olympus, Hamburg, Germany). The number of fruits used ranged from 12-60.

**Method S4 Determining fruit growth parameters**

Developing apple fruits from ‘Karmijn’, ‘Pinova’, ‘Idared’, and ‘Gala’ were collected in one (7 to 49 DAFB) to two (63 to maturity) week intervals during the 2020 growing season. Each data point represents a total of 30 fruits collected from individual trees. Fruit mass was obtained by using a digital balance (CPA225D; Satorius, Göttingen, Germany) and fruit diameters were estimated from fruit mass by assuming a spherical shape and a density of 1 kg dm^-3^. Surface area growth rate and relative surface area growth rate were determined as previously described [6].

**References**

1. Karnovsky MJ. A formaldehyde-glutaraldehyde fixative of high osmolarity for use in electron microscopy. J. Cell Biol. 1965;27:1A–149A.

2. Chen Y-H, Straube J, Khanal BP, Knoche M, Debener T. Russeting in apple is initiated after exposure to moisture ends-I. Histological evidence. Plants 2020. doi:10.3390/plants9101293.

3. Straube J, Chen Y-H, Khanal BP, Shumbusho A, Zeisler-Diehl V, Suresh K, et al. Russeting in apple is Initiated after exposure to moisture ends: Molecular and biochemical evidence. Plants 2020. doi:10.3390/plants10010065.

4. Chen Y-H, Straube J, Khanal BP, Zeisler-Diehl V, Suresh K, Schreiber L, et al. Apple fruit periderms (russeting) induced by wounding or by moisture have the same histologies, chemistries and gene expressions. PLoS One. 2022;17:e0274733. doi:10.1371/journal.pone.0274733.

5. Brundrett MC, Kendrick B, Peterson CA. Efficient lipid staining in plant material with sudan red 7B or fluorol correction of fluoral yellow 088 in polyethylene glycol-glycerol. Biotech Histochem. 1991;66:111–6. doi:10.3109/10520299109110562.

6. Khanal BP, Imoro Y, Chen YH, Straube J, Knoche M. Surface moisture increases microcracking and water vapour permeance of apple fruit skin. Plant Biol. 2021;23:74–82. doi:10.1111/plb.13178.

7. Peschel S, Knoche M. Characterization of microcracks in the cuticle of developing sweet cherry fruit. J. Am. Soc. Hortic. Sci. 2005;130:487–95. https://doi.org/10.3390/plants10010065.
