# Supplementary material for: Time course of changes in the transcriptome during russet induction in apple fruit
Source: BMC Plant Biol. 2023 Sep 30;23:457. doi: 10.1186/s12870-023-04483-6 (PMC10542230; doi:10.1186/s12870-023-04483-6)
Supplement: Supplementary file 14 — Supplementary Material 14 [file 12870_2023_4483_MOESM14_ESM.docx]

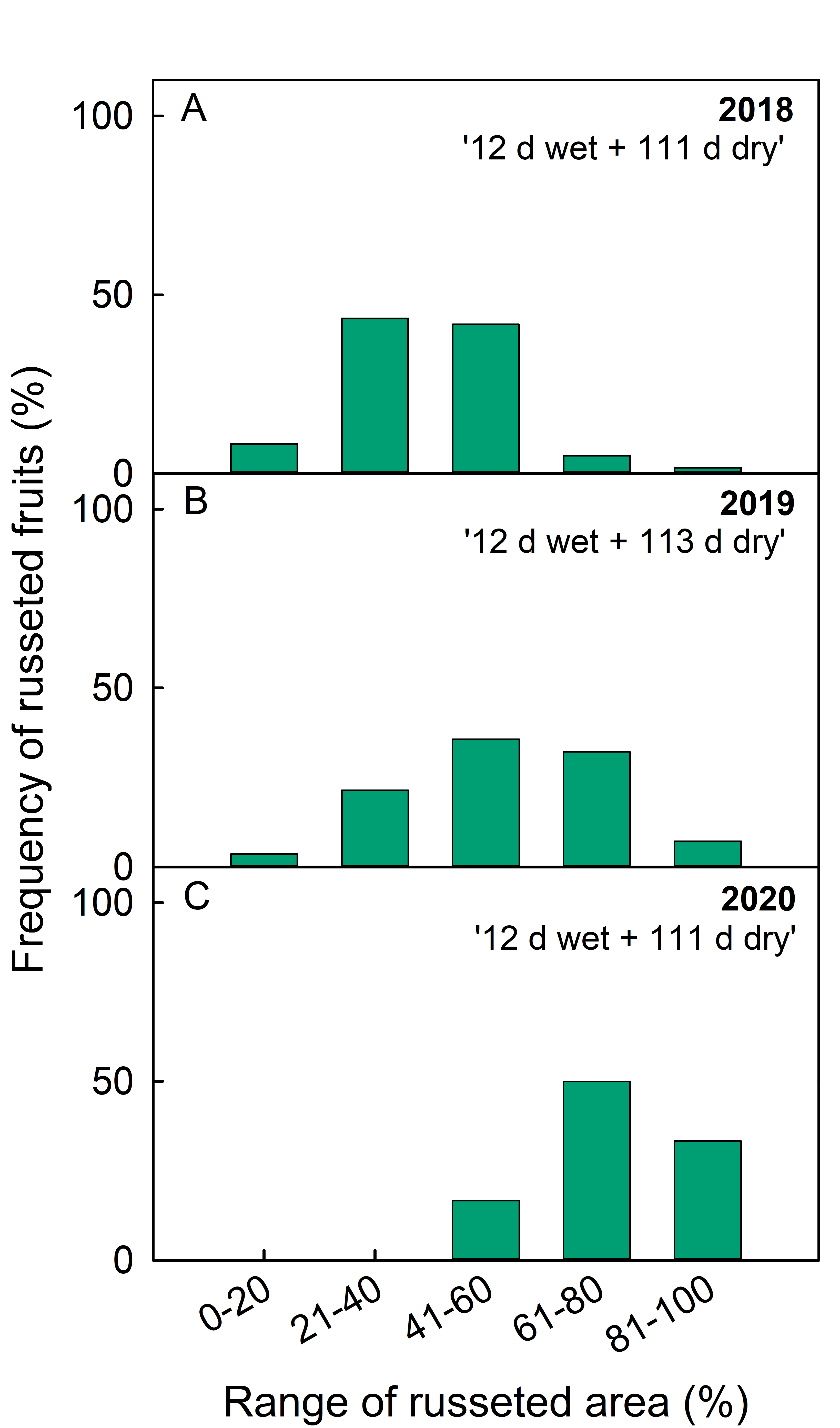


**Figure S2 Frequency distribution of russeting of ‘Pinova’ apples in the 2018 (*n* = 60) (A), 2019 (*n* = 28) (B) and 2020 growing seasons (*n* = 12) (C).** Russeting was induced by exposure to surface moisture for 12 d (Phase I) beginning at 21-31 DAFB. After termination of moisture exposure, the fruit skin patches remained dry (Phase II). The portion of the russeted surface area exposed to moisture was quantified at maturity.
