# Supplementary material for: Time course of changes in the transcriptome during russet induction in apple fruit
Source: BMC Plant Biol. 2023 Sep 30;23:457. doi: 10.1186/s12870-023-04483-6 (PMC10542230; doi:10.1186/s12870-023-04483-6)
Supplement: Supplementary file 15 — Supplementary Material 15 [file 12870_2023_4483_MOESM15_ESM.docx]

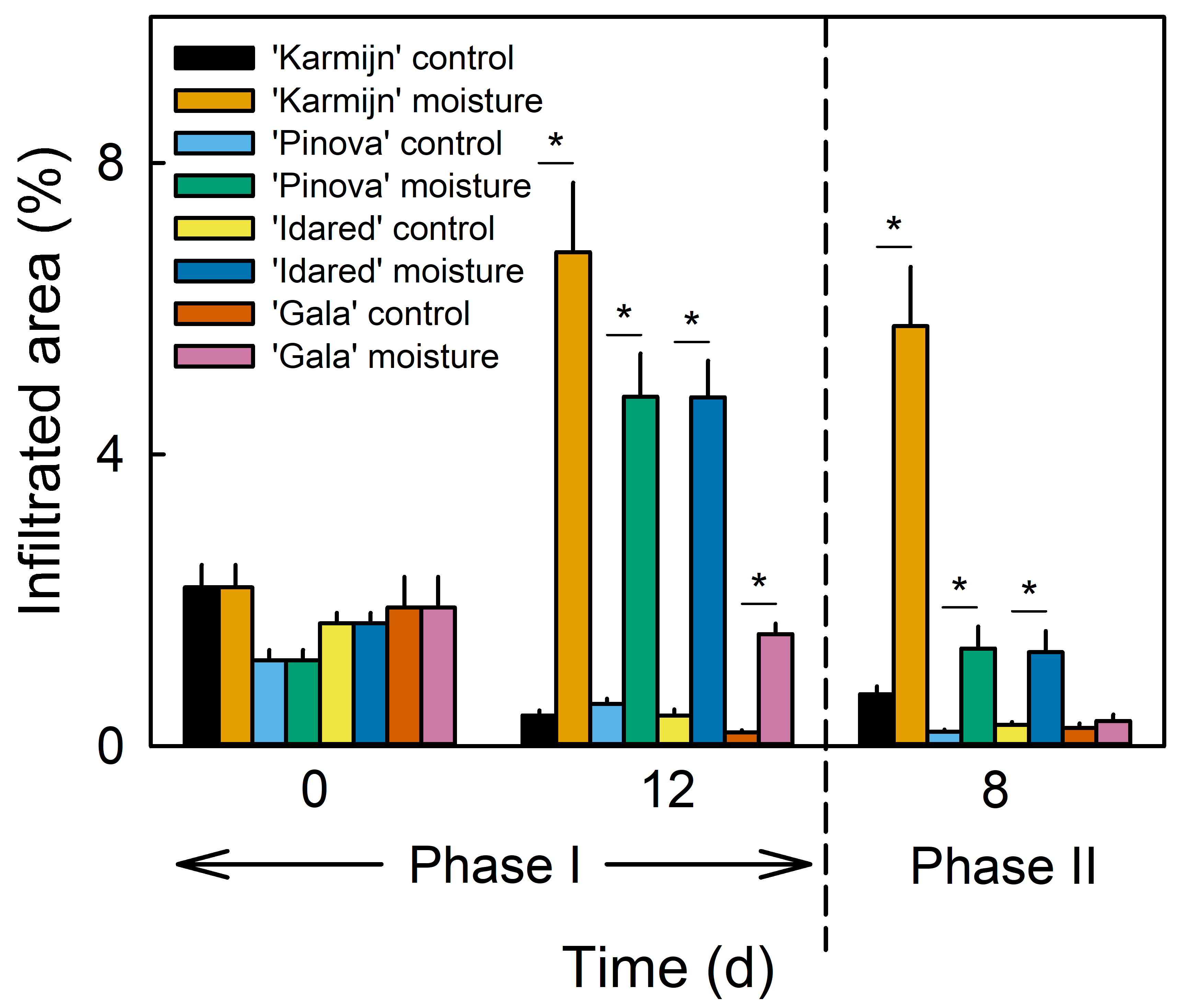


**Figure S5 Microcrack formation in the cuticle of apples in four cultivars following exposure to moisture.** Fruit skin patches of ‘Karmijn’, ‘Pinova’, ‘Idared’, and ‘Gala’ were exposed to surface moisture beginning at 28-32 days after full bloom (DAFB) for 12 d (Phase I). Afterwards, moisture was removed and the treated fruit skin patch remained dry (Phase II). The nonexposed surface of the same fruit served as a control. Microcracking was indexed by quantifying the area infiltrated with acridine orange. Data represent the means ± SEs of ten fruits. ‘*’ indicates a significant difference between ’Moisture’ and ‘Control’ within each cultivar at *p* ≤ 0.05 (Student’s t test).
