# Supplementary material for: Time course of changes in the transcriptome during russet induction in apple fruit
Source: BMC Plant Biol. 2023 Sep 30;23:457. doi: 10.1186/s12870-023-04483-6 (PMC10542230; doi:10.1186/s12870-023-04483-6)
Supplement: Supplementary file 16 — Supplementary Material 16 [file 12870_2023_4483_MOESM16_ESM.docx]

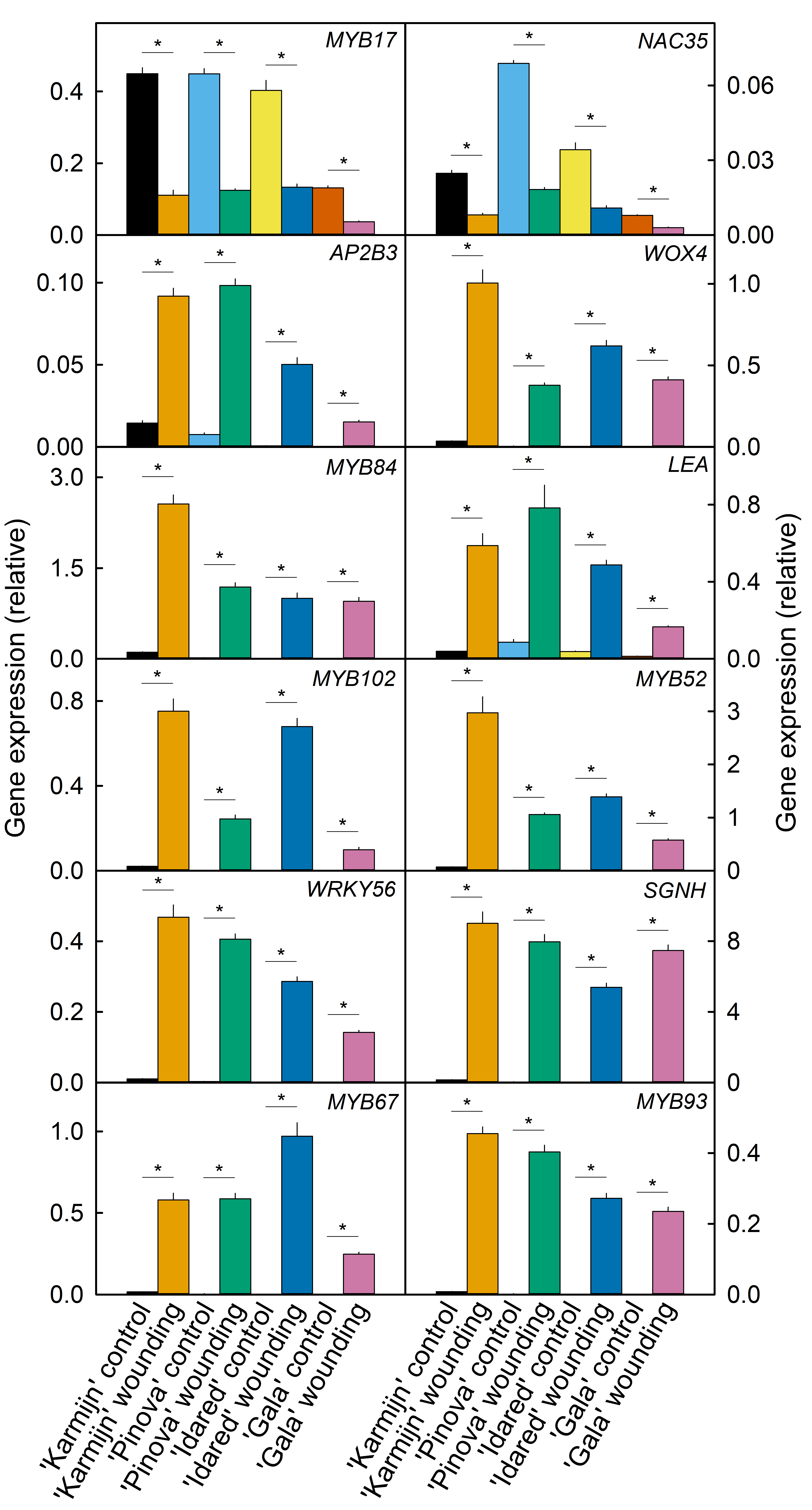


**Additional file 6: Figure S6** **Expression of candidate genes after mechanical wounding of the skin of four apple cultivars.** Russet susceptibility decreased in the order ‘Karmijn’>‘Pinova’>‘Idared’>‘Gala’. Fruit skins were wounded 38-40 days after full bloom (DAFB) using sandpaper (‘Wounding’). The nontreated fruit skin served as the control (‘Control’). Expression of genes associated with Phase I (*MYB17*, *NAC35*) or Phase II (*AP2B3*, *WOX4*, *MYB84*, *LEA*, *MYB102*, *MYB52*, *WRKY56*, *SGNH*, *MYB67*, *MYB93*) was analyzed. Expression values represent the means ± SEs of three independent replicates comprising six fruits each. ‘*’ indicates a significant difference between ‘Wounding’ and ‘Control’ within each cultivar at *p* ≤ 0.05 (Student’s t test).
