# Supplementary material for: Time course of changes in the transcriptome during russet induction in apple fruit
Source: BMC Plant Biol. 2023 Sep 30;23:457. doi: 10.1186/s12870-023-04483-6 (PMC10542230; doi:10.1186/s12870-023-04483-6)
Supplement: Supplementary file 18 — Supplementary Material 18 [file 12870_2023_4483_MOESM18_ESM.docx]

**Figure S10 Sketch of experimental design.** The moisture treatment (Moisture) experiment consisted of two phases: In Phase I (blue arrow) the fruit skin patches were exposed to moisture. In Phase II the moisture exposure was terminated and the skin patch exposed to atmospheric conditions. In the wounding experiments, the fruit skin patches were intentionally wounded by gently abrading them with sandpaper. The timing of the abrasion coincided with the end of moisture treatment in Phase I.
