# Supplementary material for: Time course of changes in the transcriptome during russet induction in apple fruit
Source: BMC Plant Biol. 2023 Sep 30;23:457. doi: 10.1186/s12870-023-04483-6 (PMC10542230; doi:10.1186/s12870-023-04483-6)
Supplement: Supplementary file 19 — Supplementary Material 19 [file 12870_2023_4483_MOESM19_ESM.docx]

**
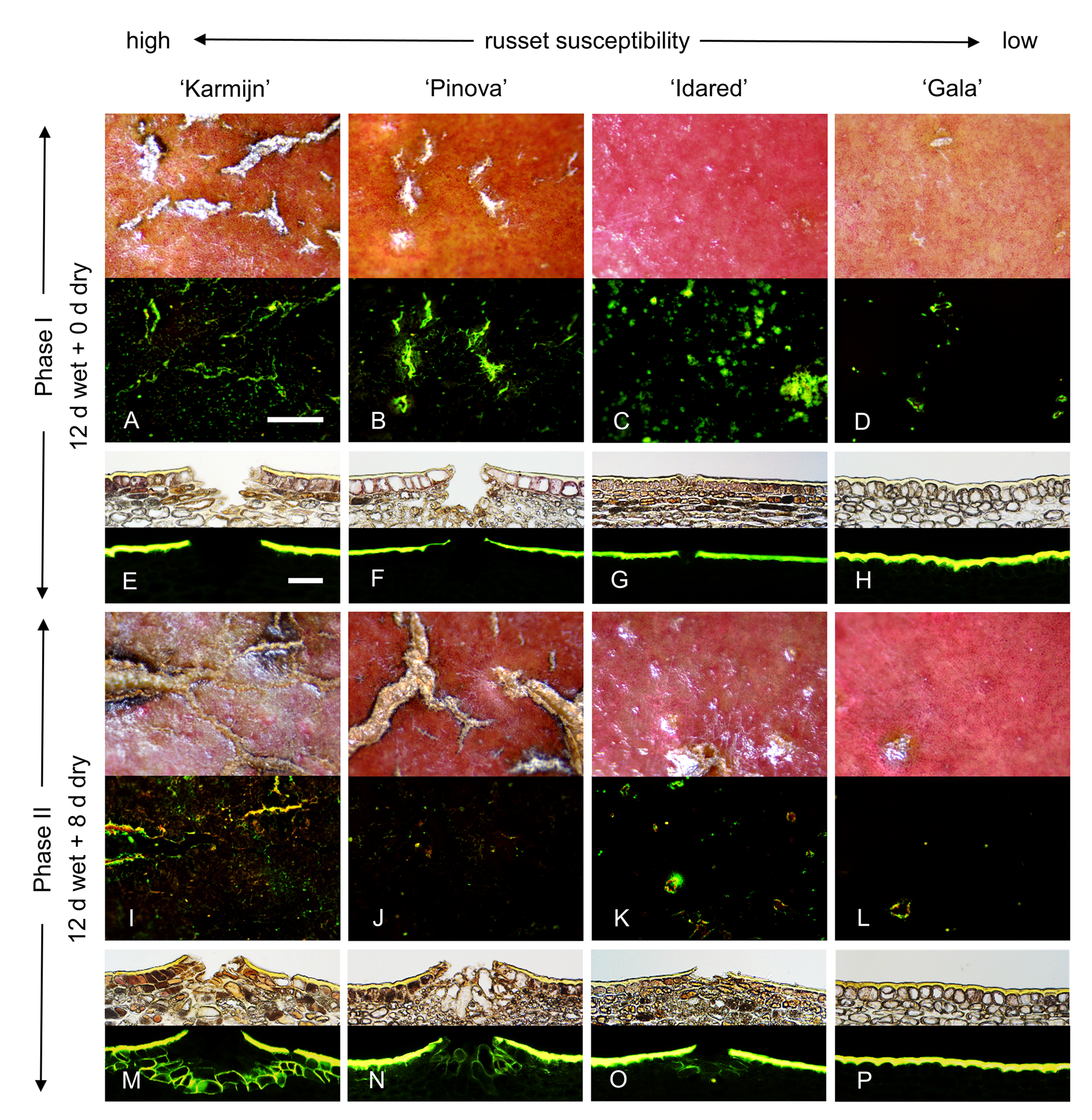
**

**Figure S3** **Comparison of microcrack and periderm formation in four apple cultivars differing in russet susceptibility.** Russet susceptibility decreased from ‘Karmijn’ to ‘Pinova’ and from ‘Idared’ to ‘Gala’. Russeting was induced in a two-phase experiment. During Phase I, skin patches of ‘Karmijn’ (A, E, I, M), ‘Pinova’ (B, F, J, N), ‘Idared’ (C, G, K, O) and ‘Gala’ (D, H, I, P) were exposed to moisture for 12 d (‘x d wet’). After termination of moisture exposure (Phase II), the treated skin patch was exposed to the ambient atmosphere for up to 8 d (‘y d dry’). The nontreated control (‘Control’) remained dry during Phase I and Phase II (‘x d dry + y d dry’).

Exposure to moisture started 28-32 days after full bloom (DAFB), equivalent to 10-12 mm diameter. Light micrographs and corresponding fluorescence micrographs of fruit surfaces after infiltration of microcracks with acridine orange (A-D, I-L). The scale bar in (A) is 200 µm long and is representative of the images in A, B, C, D, I, J, K and L (*n* = 10). Cross-sections through skin segments stained with Fluorol Yellow 088 were observed by transmitted white light (upper part) and incident fluorescent light (filter U-MWB) (E-H, M-P). The scale bar in (E) is 50 µm long and representative of the micrographs in E-H and M-P (*n* = 6).
