# Supplementary material for: Time course of changes in the transcriptome during russet induction in apple fruit
Source: BMC Plant Biol. 2023 Sep 30;23:457. doi: 10.1186/s12870-023-04483-6 (PMC10542230; doi:10.1186/s12870-023-04483-6)
Supplement: Supplementary file 20 — Supplementary Material 20 [file 12870_2023_4483_MOESM20_ESM.docx]

**
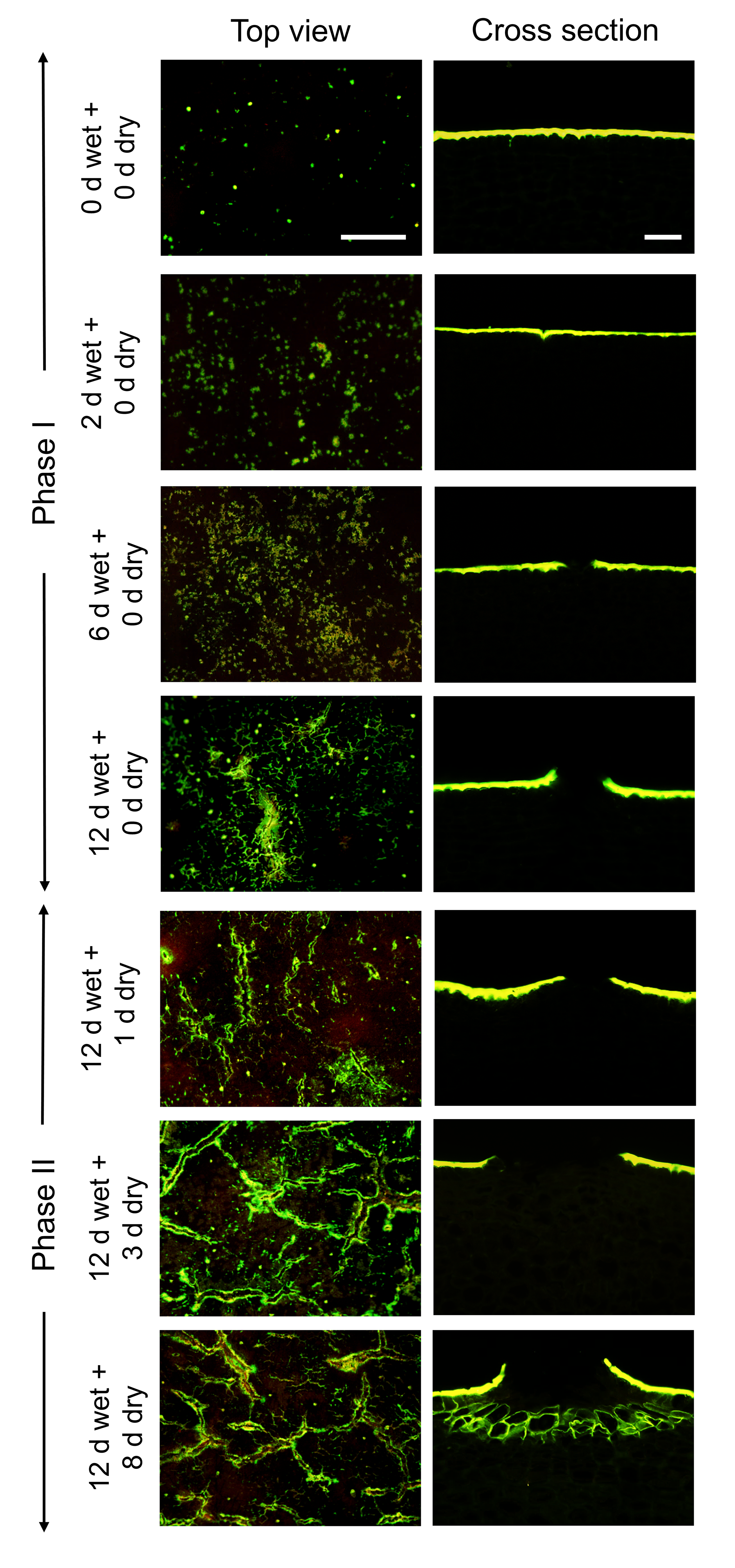
**

**Figure S1** **Time course of moisture-induced russeting of ‘Pinova’ apple as indexed by fluorescence microscopy.** Russeting was induced in a two-phase experiment: During Phase I, a patch of fruit skin was exposed to surface moisture for 12 d (Phase I, ‘12 d wet’). After termination of moisture exposure (Phase II), the treated skin patch was exposed to the ambient atmosphere (‘y d dry’). The nontreated control (‘Control’) remained dry during Phase I and Phase II (‘x d dry + y d dry’). Moisture was applied at 31 days after full bloom (DAFB). Top view (left panel) and cross-sections of the fruit surface (right panel) during formation of microcracks in the cuticle and initiation of a periderm. Microcracks were infiltrated with acridine orange (left panel), and the cross-sections were stained with Fluorol Yellow 088 (right panel). The scale bar in the top view of ‘0 d wet + 0 d dry’ is 200 µm long and representative of all top views (*n* = 10). The white scale bar in the ‘0 d wet + 0 d dry’ cross-section is 50 µm long and representative of all cross-sections (*n* = 6).
