# Supplementary material for: Time course of changes in the transcriptome during russet induction in apple fruit
Source: BMC Plant Biol. 2023 Sep 30;23:457. doi: 10.1186/s12870-023-04483-6 (PMC10542230; doi:10.1186/s12870-023-04483-6)
Supplement: Supplementary file 21 — Supplementary Material 21 [file 12870_2023_4483_MOESM21_ESM.docx]

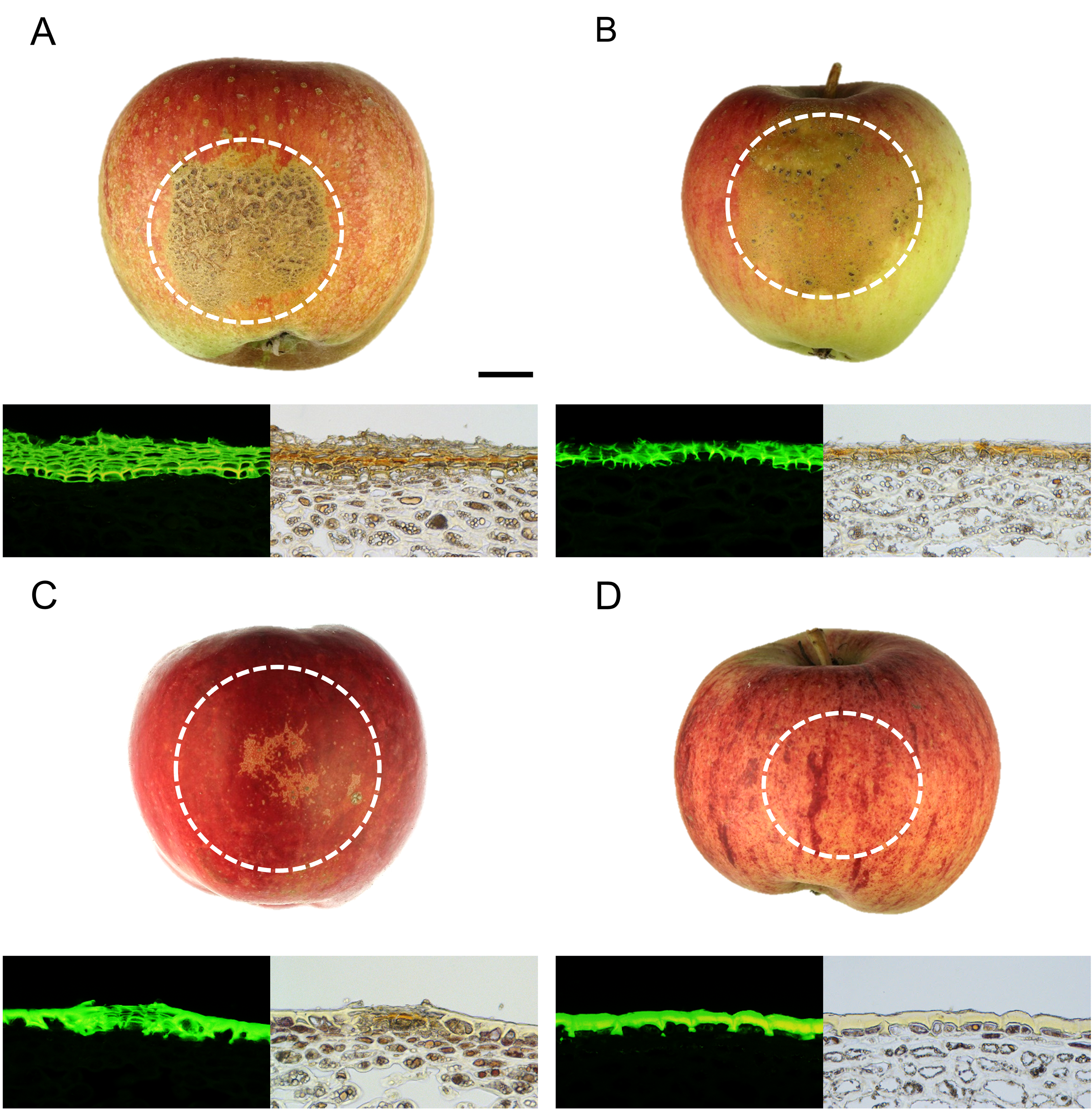


**Figure S4 Macrographs and micrographs of moisture-exposed fruit surfaces of four apple cultivars differing in russet susceptibility.** Russet susceptibility decreases in the order ‘Karmijn’>‘Pinova’>‘Idared’ > ‘Gala’. Russeting was induced in a two-phase experiment. During Phase I, skin patches of ‘Karmijn’ (A), ‘Pinova’ (B), ‘Idared’ (C) and ‘Gala’ (D) apples were exposed to moisture for 12 d. After termination of moisture exposure (Phase II), the treated skin patch was exposed to the ambient atmosphere (‘y d dry’). The nontreated control (‘Control’) remained dry during Phase I and Phase II (‘x d dry + y d dry’). Moisture exposure began at 28-32 days after full bloom (DAFB). Russeting was evaluated at maturity. Macrographs were obtained by photography of the fruit; the micrographs were prepared by fluorescence microscopy of skin sections stained with Fluorol Yellow 088 and viewed under transmitted white light or incident fluorescent light (filter U-MWB) (*n* = 3). The black scale bar in (A) is 1 cm and representative of all photographs of whole fruit. The white scale bar in (A) is 50 µm and representative of all micrographs. The white dashed circle indicates the patch of fruit skin that was exposed to moisture.
