# Supplementary material for: Time course of changes in the transcriptome during russet induction in apple fruit
Source: BMC Plant Biol. 2023 Sep 30;23:457. doi: 10.1186/s12870-023-04483-6 (PMC10542230; doi:10.1186/s12870-023-04483-6)
Supplement: Supplementary file 22 — Supplementary Material 22 [file 12870_2023_4483_MOESM22_ESM.docx]

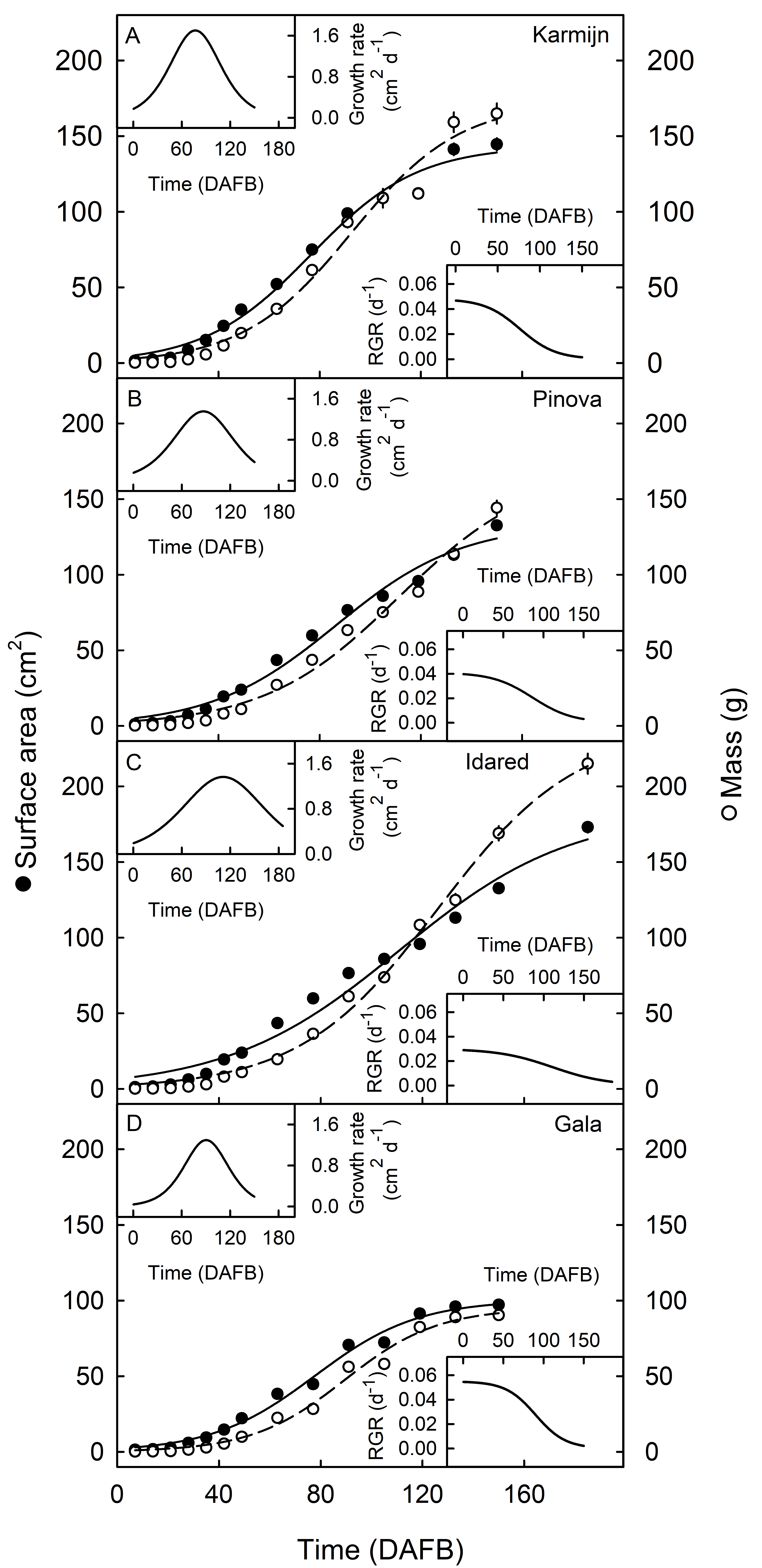


**Figure** **S8 Time course of changes in mass and surface area of apples in four cultivars.** Fruit growth parameters in ‘Karmijn’ (A), ‘Pinova’ (B), ‘Idared’ (C), and ‘Gala’ (D) were determined in the 2020 growing season. The two insets illustrate the surface area growth rate (upper left corner) and relative surface area growth rate (RGR; lower right corner) in developing apple fruits. X-axis scale in days after full bloom (DAFB). Each value represents the mean ± SE of 30 fruits.
