# Supplementary material for: Time course of changes in the transcriptome during russet induction in apple fruit
Source: BMC Plant Biol. 2023 Sep 30;23:457. doi: 10.1186/s12870-023-04483-6 (PMC10542230; doi:10.1186/s12870-023-04483-6)
Supplement: Supplementary file 23 — Supplementary Material 23 [file 12870_2023_4483_MOESM23_ESM.docx]

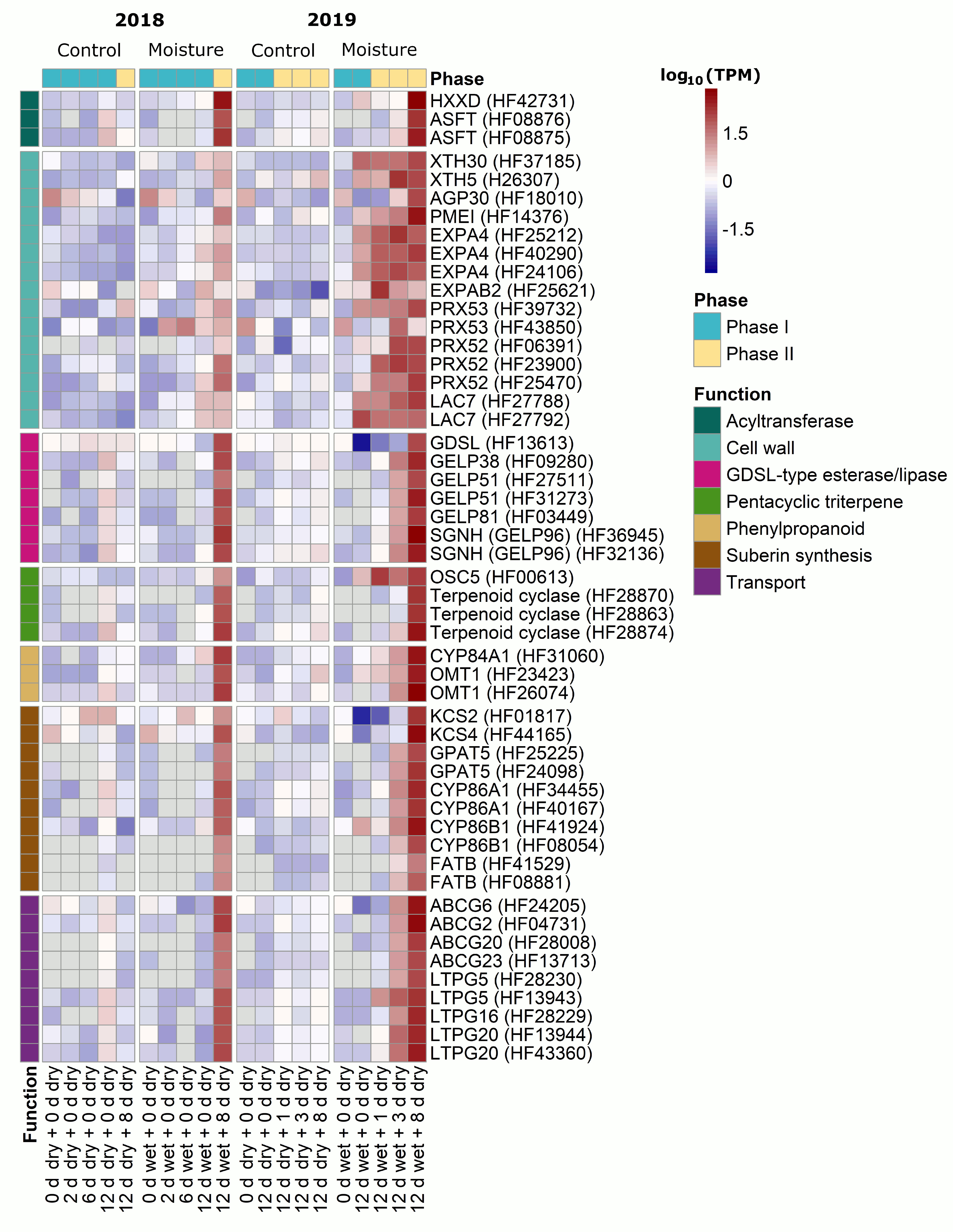


**Figure** **S9** **Heatmap of genes related to periderm formation during moisture-induced russeting in ‘Pinova’ apple.** Russeting was induced in a two-phase experiment. During Phase I, skin patches of ‘Pinova’ apples were exposed to moisture for 2, 6 or 12 d (‘x d wet’). After termination of moisture exposure (Phase II), the treated skin patch was exposed to the ambient atmosphere for 1, 3 or 8 d (‘y d dry’). The nontreated control (‘Control’) remained dry during Phase I and Phase II (‘x d dry + y d dry’). Genes associated with russeting (based on literature information) were upregulated during Phase II. Expression values are shown as the mean log_10_(TPM) (transcripts per million) values of three independent replicates comprising six (season 2018) or ten (season 2019) fruits each.
