# Supplementary material for: Time course of changes in the transcriptome during russet induction in apple fruit
Source: BMC Plant Biol. 2023 Sep 30;23:457. doi: 10.1186/s12870-023-04483-6 (PMC10542230; doi:10.1186/s12870-023-04483-6)
Supplement: Supplementary file 24 — Supplementary Material 24 [file 12870_2023_4483_MOESM24_ESM.docx]

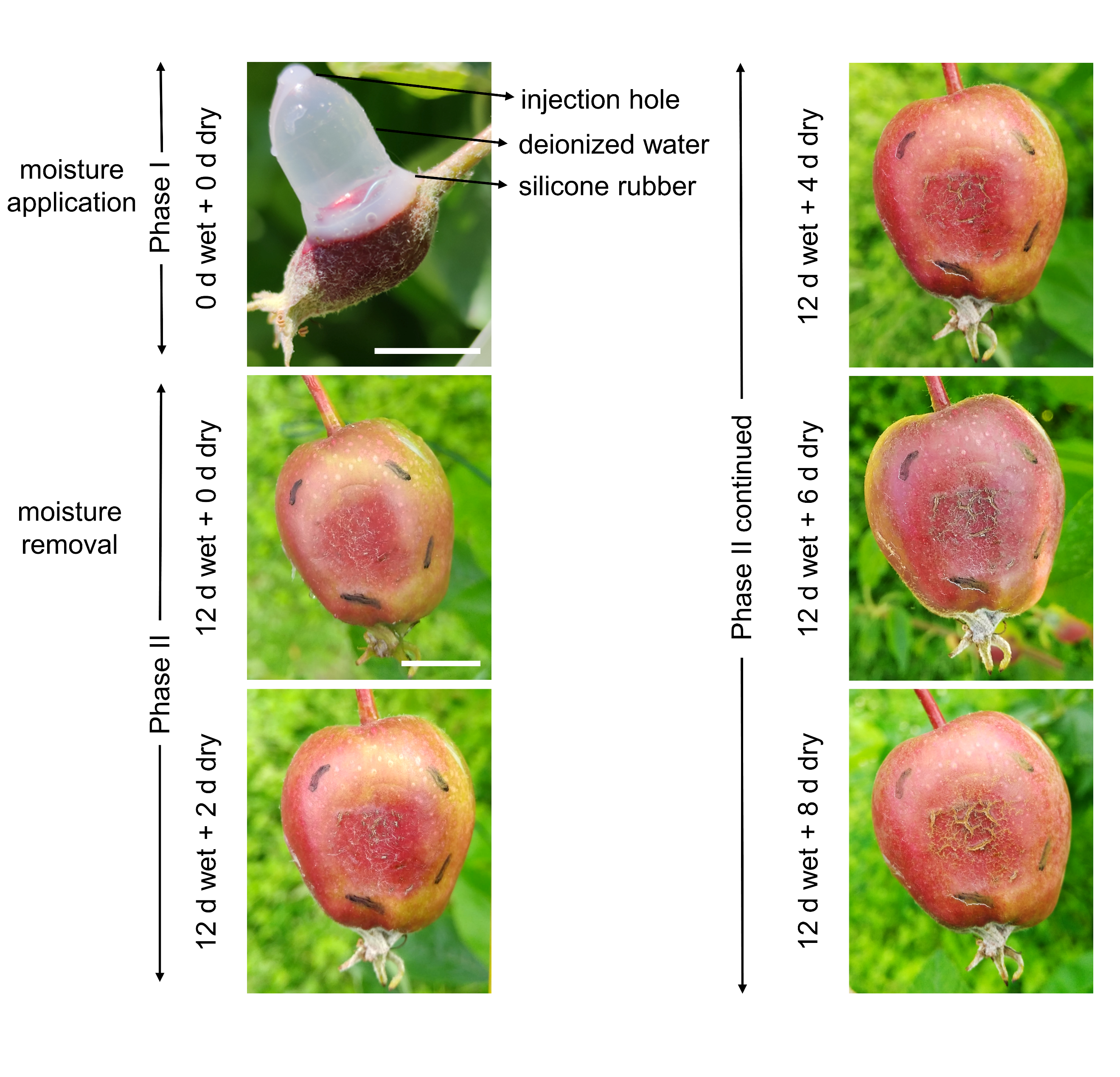


**Figure S11 Russet induction by surface moisture**. Russeting of 'Pinova' apple fruit skin was induced by applying moisture through a polyethylene tube mounted on the fruit surface using a non-phytotoxic silicone rubber during early stages of fruit development (21-31 days after full bloom (DAFB)). After curing, deionized water was injected through a hole at the top of the polyethylene tube using a syringe. The hole was sealed thereafter using silicone rubber. The fruit skin was exposed to surface moisture for a period of 12 d (Phase I). After this period, the tube was carefully removed, and the microcracked surface was exposed to atmospheric conditions (Phase II). The treated fruit skin patch was marked with a black, water-resistant marker. During Phase II, moisture-exposed fruit skins showed macroscopically visible cracks within 2 d after moisture removal (12 d wet + 2 d dry). A fully russeted skin patch formed after 12 d wet + 8 d dry. In the accompanying images, the white scale bars for '0 d wet + 0 d dry' and '12 d wet + 0 d dry' are 1 cm long, the latter being representative of all other images.
